# All-in-one optically interactive soft robots with embedded liquid crystal holography

**DOI:** 10.1038/s41377-026-02287-5

**Published:** 2026-05-06

**Authors:** Zi-Chen Zhang, Yang Wei, Ze-Yu Wang, Ying-Hao Fu, Ren Zheng, Ning Wang, Yu Wang, Ling-Ling Ma, Yan-Qing Lu

**Affiliations:** National Laboratory of Solid State Microstructures, Key Laboratory of Intelligent Optical Sensing and Manipulation, College of Engineering and Applied Sciences, and Collaborative Innovation Center of Advanced Microstructures, Nanjing University, Nanjing, 210023 China

**Keywords:** Liquid crystals, Displays

## Abstract

Soft robots capable of self-driven information transmission hold great promise for enabling intelligent interactions that better emulate the behavior of living organisms; however, achieving such systems remains elusive. Here, we present an all-in-one optically interactive soft robot that seamlessly integrates holographic command encoding, encryption, and display with on-demand task execution. By leveraging the unique combination of liquid crystal and silk fibroin, this system achieves a synergistic integration of multi-degree-of-freedom actuation and information multiplexing within an all-soft-matter modular architecture. This “information-machine” coupling paradigm encodes task instructions into encrypted holographic feedback, ensuring the reliable execution of complex operations only upon accurate decoding. As demonstrations, we showcase an intelligent gripper capable of precise object grasping and classification in response to decoded holographic commands, as well as a walking robot that navigates a maze guided by multi-level, decrypted holographic pathways. The proposed strategy establishes a new framework for developing interactive soft robots that closely mimic living organisms by employing light as a central information carrier.

**Figure:**
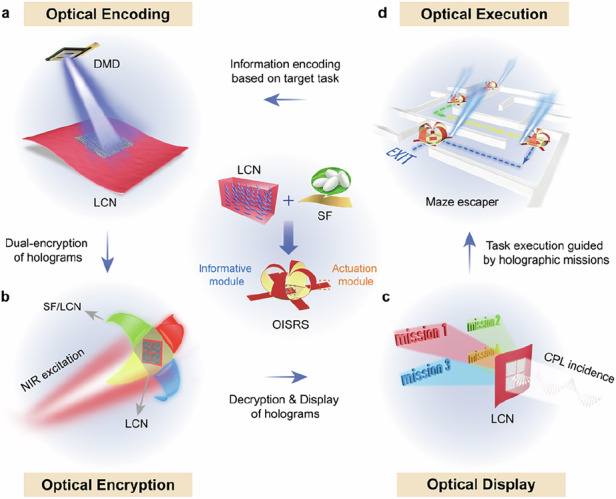
**Short summary** We describe an optically interactive soft robotic system (OISRS) that utilizes LC computational holography for embedded optical command processing and decision-making

**Short summary** We describe an optically interactive soft robotic system (OISRS) that utilizes LC computational holography for embedded optical command processing and decision-making

## Introduction

Soft robotics has emerged as a transformative technology in the field of robotics, characterized by high degrees of freedom, exceptional continuous deformability, superior environmental adaptability, and safe human-robot interaction^1–4^. Unlike traditional rigid robots, soft robots can better emulate the compliance and adaptive behaviors of biological organisms, offering significant potential in applications such as human-robot interaction, precision medicine, disaster rescue, deep-sea exploration, and space missions^5–8^. Recent advances in material engineering and structural programming have enabled soft robots to achieve diverse three-dimensional (3D) morphing and complex, multimodal locomotion, supporting the execution of sophisticated tasks, including navigation in complex environments^9–11^, adaptive grasping and object manipulation^12–14^, as well as minimally invasive surgery and targeted therapy^15–17^. The successful execution of these tasks by operators fundamentally involves two key elements: defining what tasks to perform (command) and determining how to perform them (actuation). Particularly in mission-critical applications, soft robots can only fulfill their tasks when operated precisely according to the encoded command. However, existing soft robotic systems remain dependent on either external hardware-based control architectures or human cognitive input for task command generation and interpretation^18–21^, while the robots themselves lack the integrated capabilities for information storage and transmission, consequently rendering them unable to guide the operator on what tasks to perform and how to perform them. This critical limitation stems from the absence of an embedded information storage unit analogous to a biological neural center, which can send precise instructions for human movement guidance, thus preventing the realization of localized information encoding, processing, and transmission within the robotic system itself. Consequently, this restricts their task adaptability and operational reliability in complex scenarios. Therefore, developing an all-soft robotic architecture with an embedded information management center for operator guidance represents a crucial pathway toward enhancing its interactivity and autonomy.

Soft-matter holography is an emerging technology that integrates the dynamic tunability of soft materials^22,23^ (e.g., liquid crystals (LCs), polymers, and biomaterials) with holographic information encoding^24^. Conventional soft-matter holographic systems primarily employ refractive index grating modulation^25–28^. Recently, geometric-phase-based liquid crystal (LC) holography has garnered significant attention due to its unique advantages, including tunable operation and broadband spectral adaptability^29,30^. By harnessing the intrinsic birefringence and optical phase modulation capabilities of LC molecules, this approach enables dynamic wavefront modulation for hologram generation. Recent advancements of LC holography have demonstrated its remarkable progress in high-density optical information encoding and storage, dynamic holographic display, and multi-channel encryption/processing^31–33^. Incorporating this technology into soft robotic systems offers a promising route to non-electronic, high-density, and secure information storage and processing, thereby advancing the development of soft robots toward more sophisticated, bio-inspired intelligent systems. Specifically, by designing LC superstructure-based hologram interfaces, soft robots could on-demand store precise task commands or environmental mapping information and enable human-robot interaction via optical feedback, thus effectively addressing the current limitations in onboard information processing within soft robotic platforms.

In this work, we describe an optically interactive soft robotic system (OISRS) that utilizes LC computational holography for embedded optical command processing and decision-making. This soft robotic system is constructed using an all-soft-matter modular architecture primarily composed of liquid crystal networks (LCNs) and silk fibroin (SF). We demonstrate that the unique combination of LCN and SF enables both (i) multi-degree-of-freedom actuation through their programmable responses to light and humidity, respectively, and (ii) information multiplexing via their capacity for multimodal encoding. We establish that this system creates a closed-loop framework for integrating command encoding, data encryption, visual display, and on-demand execution, facilitating the reliable execution of specialized, complex tasks. Such polymer-film devices, which enable optical interaction of command information through holographic imaging, serve in our system as an information-integration unit analogous to the central nervous system in biological organisms, thus offering a promising approach to addressing the limitations in current soft robotic systems, as stated above. As proof of concept, we showcase an intelligent gripper capable of precise, on-demand grasping and object classification guided by an integrated holographic instruction. Finally, we demonstrate the successful navigation of a walking robot through a maze, following a multi-level encrypted holographic pathway.

## Results

### Concept of OISRS

**Fig. 1:**
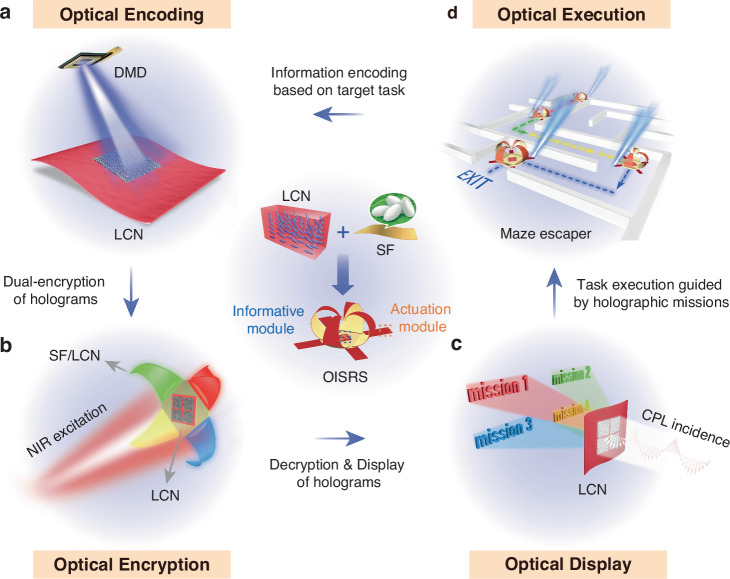
Schematic illustration of the OISRS design. The system integrates the following functionalities: (**a**) information encoding, (**b**) encryption, (**c**) display, and (**d**) execution.The LCN-SF composite synergistically combines multi-degree-of-freedom actuation with integrated information processing capabilities

The OISRS integrates actuation and information functionalities within a unified architecture by employing a liquid crystal network/silk fibroin (LCN/SF) bilayer as the actuation module and an encrypted LCN film with embedded holograms as the informative module. During operation, the operator decodes task commands embedded within the informative module and, guided by this visual feedback, precisely controls the actuation module to execute corresponding tasks. Figure 1 illustrates the closed-loop design of the OISRS. In this system, the intrinsic photoalignment capability of LCs is exploited to record phase holograms using a digital micromirror device (DMD)-based micro-lithography system, thereby encoding task-specific optical information directly into a solid-state LCN film (Fig. 1a). Leveraging the exceptional shape programmability and dopant-hosting ability of SF, the morphing SF layer, doped with upconversion nanoparticles (UCNPs), provides both three-dimensional architecture and near-infrared (NIR)-excitable fluorescence for optical encryption (Fig. 1b). Following predefined decryption scheme, illumination with circularly polarized light (CPL) reconstructs distinct holographic images from the LCN layer, which exhibit a one-to-one correspondence with the fluorescence colors of the SF layer. This correlation enables reliable information extraction and multidimensional optical display (Fig. 1c). The decoded holographic images thus act as optical command interfaces, allowing operators to interpret encoded instructions and execute tasks with precise guidance (Fig. 1d).

The LCN holography we developed enables, for the first time, the creation of liquid-crystal-polymer solid-state films with embedded phase holograms. In contrast to conventional holographic systems that rely on fluidic liquid crystalline states, this solid-state configuration enables efficient integration with soft robotic platforms through interfacial assembly. Within the OISRS, the holographic LCN serves as an information-integration unit—functionally analogous to the central nervous system in biological organisms—where holographic feedback dynamically mediates optical information exchange between the operator and the robotic system.

Furthermore, the synergistic coupling between the LCN and SF layers confers multifunctionality that neither material could achieve independently. Specifically, LCNs possess excellent photothermal responsiveness and holographic encoding capacity, yet their limited thermoplasticity, single-mode actuation behavior, and incompatibility with functional dopants restrict their versatility in soft robotic systems. In contrast, SF films exhibit outstanding morphability, humidity-driven actuation, and high dopant compatibility, effectively compensating for the intrinsic limitations of LCNs. As such, the integration of these two materials yields a multifunctional platform that enables both multi-degree-of-freedom actuation and information multiplexing. This material synergy establishes a robust materials platform for constructing all-in-one soft robotic systems that unify information encoding, encryption, display, and actuation within a single optically interactive framework.

### Dual-responsive LCN/SF bilayer actuation module

**Fig. 2:**
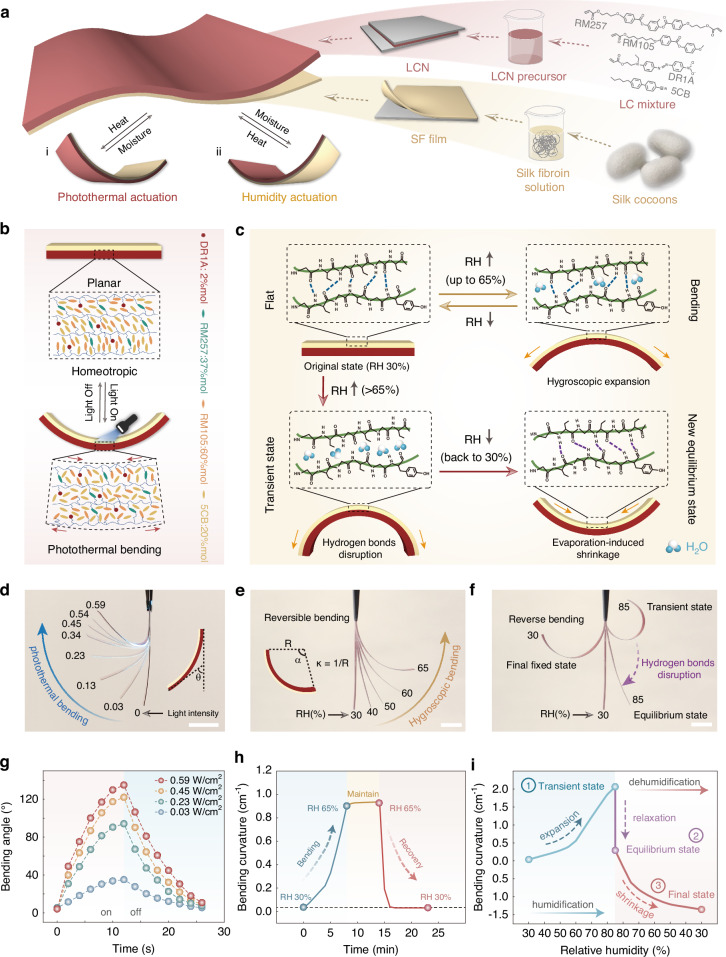
Structure, mechanism, and actuation performance of the LCN/SF bilayers. **a** Schematic illustration of material composition and actuation principle in the photothermal radiation state i) and high humidity state ii). **b** Photothermal actuation mechanism of the LCN layer. Light irradiation induces reversible switching between the splayed and disordered molecular configurations. **c** Humidity-triggered actuation mechanism of the SF layer. The flat bilayer film shows hygroscopic expansion when the RH increases to a level below 65% and reverts to its original flat state upon returning to 30% RH. However, when RH exceeds 65%, it shows transient bending due to the disruption of hydrogen bonds by water molecules, and subsequently bends toward the SF layer side upon removal of the high humidity. **d** Digital photos of the bilayer actuator under different light intensities. *θ* represents the bending angle. Scale bar: 5 mm. **e** Digital photos of the bilayer actuator under different RH (below 65%). *κ* represents the bending curvature. Scale bar: 5 mm. **f** Digital photos of three critical states during and after exposure to 85% RH: the peak transient state, equilibrium state, and final fixed state. Scale bar: 5 mm. **g** Dependence of bending angle on the light illumination time under different intensities. **h** Time-dependent variation in bending curvature during cyclic switching between 30% and 65% RH. **i** Evolution of bending curvature during the increase of humidity from 30% to 85% RH, holding at 85% RH, and subsequently returning to 30% RH

The actuation module of the OISRS is a bilayer structure capable of dual-mode, programmable actuation in response to photothermal and humidity stimuli. Specifically, the bilayer actuator consists of an LCN and an SF film, as illustrated in Fig. 2a. The LCN film, made from a reactive mesogen mixture with a photothermal dopant and a nematogenic solvent, functions as a photo-responsive layer with a splay structure that provides alignment-asymmetry-induced highly responsive actuation to light^34–37^. The SF film, produced from regenerated *Bombyx mori* SF solution, is selected as the humidity-responsive layer due to its good hygroscopicity (Fig. S1), flexibility, ease of functionalization, favorable optical properties, and polymorphic features that enable shape programmability^38–40^. Additionally, the SF film’s negative coefficient of thermal expansion^41,42^ enhances its compatibility with LCN film, enabling improved photothermal actuation performance. The significant differences in thermal expansion and hygroscopic properties between the LCN and SF layers enable reversible, asymmetric actuation in response to moisture-photothermal stimuli. Under light exposure, the LCN layer undergoes photothermal bending toward the SF layer (Fig. 2a-i), while in high-humidity environments, the SF film exhibits synergistic hygroscopic swelling at the micro- and nanoscale, causing it to bend toward the LCN layer (Fig. 2a-ii).

The bilayer structure is fabricated by laminating the SF film onto the surface of the LCN substrate using a thin, optically transparent adhesive interlayer. This design ensures both robust interfacial bonding and mechanical stability during cyclic actuation. The as-obtained LCN/Silk bilayer actuator exhibits high mechanical flexibility (Fig. S2a). Cross-sectional SEM analysis (Fig. S2b) shows that the SF layer is tightly attached to the LCN layer, confirming the formation of a stable interface.

Figure 2b, c illustrate the underlying molecular mechanisms governing the bilayer’s actuation under photothermal and hygroscopic stimuli, respectively. The LCN layer features out-of-plane orthogonally oriented LC directors at its opposing surfaces. When photothermally heated above its glass transition temperature ( ~ 21 °C, Fig. S3), the LCN exhibits progressive disordering of the LC mesogen orientation. This leads to elongation at the homeotropic alignment (HA) surface and contraction at the planar alignment (PA) surface (Fig. S4), generating a bending moment that acts toward the PA surface (or SF layer side). To confirm that the heat-induced transition corresponds to a glass transition rather than a nematic-to-isotropic phase transition, we performed X-ray diffraction (XRD) measurements on the LCN at different temperatures (Fig. S5). The XRD patterns of LCN samples at different temperatures consistently display a narrow and sharp diffraction peak in the 18–21° range, confirming the presence of a nematic liquid crystalline phase^43–45^. Of particular significance, within the temperature range of 30–150 °C, no new diffraction peaks appear, nor do the existing peaks exhibit notable disappearance or positional shifts. These observations indicate that the crystal structure remains unchanged during heating, thereby confirming that no nematic-to-isotropic phase transition occurs within this temperature range. Upon cooling, the mesogens revert to their original alignment, restoring the bilayer to its initial state. For optimal performance, the SF film was consistently bonded to the PA surface of the LCN, unless otherwise noted, which enhanced photothermal-mechanical conversion and ensured bi-directional actuation capability. During the humidification process, the system exhibits two distinct actuation behaviors. When the relative humidity (RH) is increased to a level below the glass transition threshold of SF ( ~ 68% RH at ~30 °C in our case, Fig. S6)^46^, the SF film rapidly absorbs water and undergoes only volumetric expansion (without disruption of hydrogen bonding), causing the bilayer structure to bend toward the LCN side. When the relative humidity returns to its initial level, the structure reverts to its original flat state. However, when the RH exceeds the glass transition point, water molecules disrupt the hydrogen bonds between SF chains by interacting with their polar groups^26^. This results in chain softening and rearrangement, partially dissipating internal stresses generated by swelling. Consequently, the bilayer initially undergoes significant bending toward the LCN side (due to volumetric swelling) and then retracts to an equilibrium curvature (due to stresses dissipating). Upon removal of the high humidity, continuous evaporation of water molecules causes the bilayer to spontaneously bend toward the SF layer side, eventually reaching a new equilibrium state. This unique humidity-responsive behavior of SF films enables the 3D shape morphing of bilayer structures, laying a foundation for the development of integrated soft robots with complex configurations.

Following these mechanisms, we quantitatively evaluate the bidirectional actuation performance of bilayer actuators under light and humidity stimuli, using rectangular strips (18 mm × 2 mm). Consistent with the heating results (Fig. S7), the strip bilayer actuator exhibits rapid and pronounced photo-induced deformation when exposed to blue laser (*λ* = 488 nm) irradiation, with the deformation increasing as the light intensity rises (Fig. 2d and Movie S1). This photoinduced deformation is fully reversible across a range of light intensities, with the actuation response rate enhancing as light intensity increases (Fig. 2g). The humidity-responsive behavior of the strip bilayer actuator was evaluated under varying RH conditions. When the target RH is below 65%, the deformation of the bilayer actuator increases with rising humidity (Fig. 2e). Upon returning to 30% RH, the bilayers fully recover their original flat state (Fig. 2h). In contrast, when the target RH exceeds 65%, the bilayers exhibit irreversible reverse bending instead of complete recovery, and the residual deformation curvature increases with the previously applied humidity level (Fig. S8). To further validate this unique humidity responsiveness, we monitored the bending behavior as the RH increased from 30% to 85% (Fig. 2f and Movie S2). At 85% RH, the bilayer undergoes stress relaxation due to the disruption of hydrogen bonds, transitioning from its peak deformation state (transient state) to an equilibrium configuration (Fig. 2i). Upon removal of humidity, the film exhibits pronounced reverse bending to reach its final fixed state. During this process, the deformation retraction caused by the rearrangement of SF chains prevents the complete evaporation of excess moisture within the SF film as the bilayer returns to its initial undeformed state. Consequently, as moisture continues to evaporate, further shrinkage of the SF film drives bending toward the SF side until all moisture is completely removed. At this point, new hydrogen bonds spontaneously reorganize within the SF film, stabilizing the system in a final fixed state.

The actuation performance of this bilayer actuator exhibits strong geometric dependence, particularly on the individual layer thicknesses. (Fig. S9a, b). When the thickness of the SF layer is fixed at 30 μm, an increase in the LCN layer thickness from 30 μm to 70 μm results in a gradual reduction in the actuator’s photo-induced bending capability, likely due to higher bending stiffness and reduced thermal conduction efficiency. Conversely, when the LCN layer thickness is fixed at 50 μm, increasing the SF layer thickness from 10 μm to 50 μm enhances the water molecule adsorption and the degree of hygroscopic swelling, thereby improving the actuator’s humidity-induced bending response. All bilayer actuators in this study consist of a 50 μm LCN layer and a 30 μm SF layer unless otherwise specified. Furthermore, the bilayer actuator demonstrates high reversibility and long-term stability under both light and humidity stimuli. The photo-induced actuation maintains a consistent bending angle with no significant degradation after 100 cycles of light-on/off switching. Similarly, the humidity-responsive deformation remains stable over 100 cycles when the RH is repeatedly cycled between 30% and 65% (Fig. S9c).

**Fig. 3:**
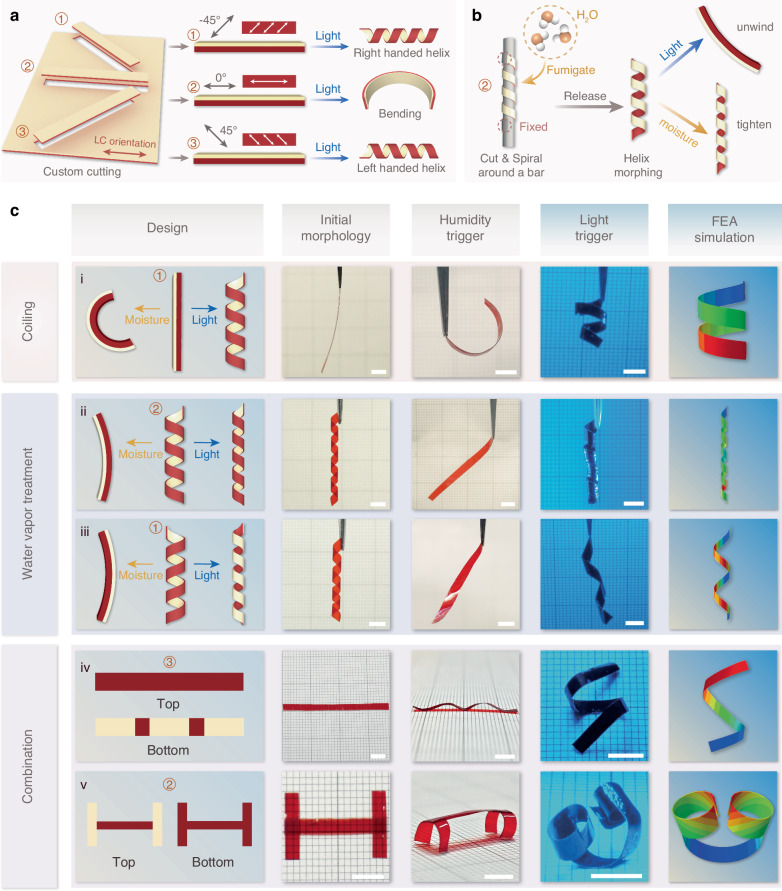
Programmable actuation performance of the bilayer actuators. **a** Programmable deformation behavior of the bilayer actuator sheared through different LC alignments. **b** Moisture-induced shape-morphing capability of the LCN/SF bilayer. **c** Schematic illustrations, experimental observations, and finite-element simulation results of five actuator configurations under light and humidity stimulation: (i) a strip cut at a 45° angle relative to the LC orientation; (ii) a spiral structure with an inner silk layer and outer LCN layer cut at 0°; (iii) a spiral with the SF film affixed to the HA surface of LCN cut at a 45° angle; (iv) an LCN strip cut along the 45° direction with three SF patches attached at regular intervals on the HA surface; and (v) an H-shaped structure formed by vertically attaching two bilayer actuators to the ends of a horizontal LCN strip. Light intensity: ~ 0.25 W/cm^2^. Scale bars: 5 mm

While the differential responsiveness of SF and LCN layers enables compelling bi-directional actuation behavior, the ability to modulate their internal molecular orientation and arrangement offers a significant opportunity for achieving programmable, multi-degree-of-freedom actuation in this bilayer system. We have provided a comparison table to showcase the functional advantages of combining these two materials (Table S1). Directional cutting of LCNs to modify the orientation of LC domains on the PA surface presents a simple yet effective strategy to enable multimodal actuation. Figure 3a illustrates how bilayer structures can be cut at different angles to achieve light-activated bending or coiling actuation modes. As demonstrated in Fig. 3c-i, cutting a strip from the bilayer at a 45° angle relative to the LC orientation induces coiling deformation under light illumination, but bending deformation under humidification. Beyond engineering molecular orientation of LCN, the moisture-induced molecular rearrangement and the resulting shape-morphing capability of SF films^26^ compensate for the inherent limitation of LCNs to undergo thermoplastic deformation, thereby enabling the design of actuators with 3D initial configurations (Fig. 3b). To demonstrate this, we shaped the bilayer into a spiral structure with two different configurations. In the first case, we cut a strip along the parallel orientation direction of the LCN and shaped it into a spiral structure, with the SF layer positioned as the inner layer and the LCN as the outer layer. During humidification, the SF film expands, causing the spiral to unwind and unfold. Conversely, laser irradiation induces LCN bending, causing the spiral to contract and tighten (Fig. 3c-ii), yielding a stimulus-responsive deformation opposite to that in Fig. 3b. In the second case, the configuration is further modified by attaching the SF film to the HA surface of LCN, and a strip was cut following the same method as in Fig. 3c-i. This strip was then morphed into a left-handed helix, with the SF film as the inner layer and the LCN as the outer layer (Fig. 3c-iii). Under humidification, the structure unwinds and expands, while under irradiation, it reversely curls into a right-handed helix.

In addition to encoding molecular structure, incorporating modular assembly strategies can enable even more complex deformation behaviors. As shown in Fig. 3c-iv, a LCN strip was cut along the 45° direction, followed by the attachment of three SF films at regular intervals onto the HA surface of the LCN. Upon humidification, the strip exhibits a “wave-like” deformation, while irradiation induces an “S”-shaped configuration. Another example is an H-shaped structure constructed by pasting two bilayer actuators vertically at the ends of a horizontal LCN strip (Fig. 3c-v). The assembled actuator could transform into a 3D “arch bridge” shape after humidification and a reversed 3D “headphone-like” shape under laser irradiation as a result of the coordinated motion of the multiple units.

These findings demonstrate that the hierarchical integration of molecular-level structure encoding and macroscale modular assembly can substantially expand the actuation degrees of freedom in this bilayer system. To gain a better understanding of the actuation behavior of the bilayer under light field stimulation, we conducted finite element analysis (FEA) to verify the various deformation modes exhibited by the bilayers. Across all examples, the actuation behaviors predicted by FEA closely align with experimental observations, underscoring the accuracy and reliability of FEA in capturing and replicating the actual behavior of the bilayer during the light-induced deformation process (Movie S3).

### Optically informative module

**Fig. 4:**
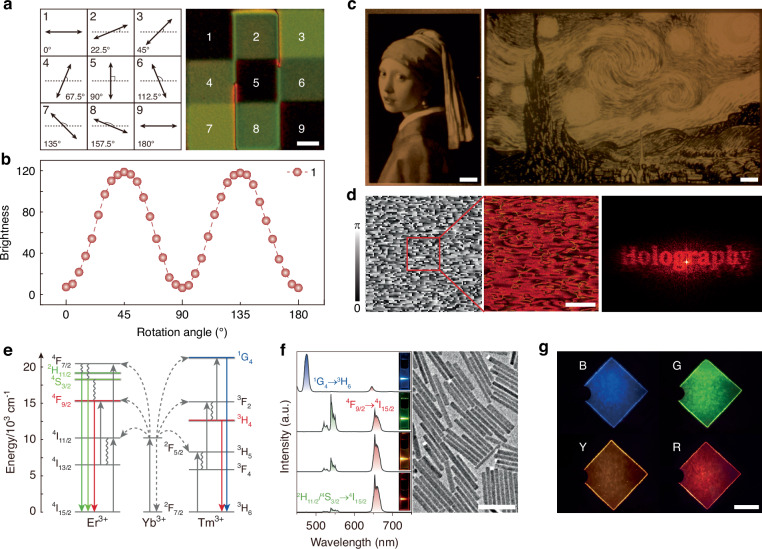
LC superstructure manipulation and multicolored UCNPs- decorated SF. **a** Design of the LC director distribution in each square of a “3 × 3” grid pattern and its corresponding POM texture. Scale bar: 20 μm. **b** Brightness variation in square 1 as a function of the sample rotation angle. **c** POM textures of artworks *Girl with a Pearl Earring* and *The Starry Night*. Scale bars: 100 μm. **d** The LC director distribution, POM texture, and holographic image corresponding to the letter “Holography”. Scale bar: 100 μm. **e** Energy level structures of rare-earth ions Er³⁺, Yb³⁺, and Tm³⁺, exhibiting tunable upconversion luminescence. **f** (Left) Photoluminescence (PL) spectra of UCNPs under 980 nm NIR laser excitation. Inset: the corresponding fluorescent photographs. (Right) Transmission electron microscopy image of NaYF_4_ nanorods. Scale bar: 200 nm. **g** UCL photos of UCNPs-doped SF films under 980 nm NIR laser excitation. Scale bar: 5 mm

LCs are widely recognized for their ability to dynamically manipulate their supramolecular architectures, leading to unique anisotropic optical properties^47–52^. To demonstrate this, we designed a “3×3 grid” pattern in which the LC directors in squares 1 through 9 rotate sequentially by increments of 22.5° (spanning 0° to 180°) using our DMD system, which enables precise spatial modulation of the LC director configuration^53–58^. In this configuration, the initial directors in squares 1, 5, and 9 are either parallel or perpendicular, those in squares 3 and 7 are oriented at 45°, and the directors in squares 2, 4, 6, and 8 are deflected by integer multiples of 22.5°, representing intermediate states. Consequently, under orthogonal polarization, the initial state of squares 1, 5, and 9 appears completely black, squares 3 and 7 exhibit maximum brightness, and squares 2, 4, 6, and 8 display intermediate brightness levels (Fig. 4a). As the sample rotates, the brightness of each square varies, with the brightness of square 1, for instance, following a sinusoidal curve over the 0°-180° range, as depicted in Fig. 4b. Building upon this design framework, we demonstrate the generation of complex optical patterns, such as famous artworks *Girl with a Pearl Earring* and *The Starry Night*, as shown in Fig. 4c. Obviously, the patterns are clearly defined in black and white, with smooth and flowing lines. Therefore, benefiting from the excellent microstructure manipulation ability of LCs, LCN holography can be developed by exposing phase holograms onto LCN film using the DMD-assisted photopatterning. The resulting holographic image is observed with our experimental setup (Fig. S10). A flow chart of the Gerchberg-Saxton algorithm for LCN holography is detailed in Fig. S11. The detailed explanations of the image-forming principle of holography, the mechanism of LCNs with embedded holograms for holographic imaging, and the DMD-assisted photopatterning process of LCNs are provided in Notes S1–S3. Figure 4d illustrates the LC director distribution, POM texture, and LCN hologram corresponding to the word “Holography”, highlighting the unique capability of LCs to encode complex microstructural patterns into on-demand, high-fidelity optical information.

Meanwhile, the incorporation of the SF layer not only enables multimodal actuation but also offers complementary optical functionalities to the OISRS. Its inherent dopant-host capacity, facilitated by aqueous processing, permits precise incorporation of photoactive species for programmable optical responses. UCNPs possess a unique combination of narrow emission bandwidth, long fluorescence lifetimes, superior photostability, and excellent biocompatibility that make them exceptionally advantageous for photonic applications^59–61^. Benefiting from the rich energy level structures of rare-earth ions, their upconversion luminescence (UCL) colors can be precisely tuned by regulating dopant types and concentrations. As shown in Fig. 4e, in Yb^3+^/Er^3+^ co-doped systems, Er^3+^ ions sequentially absorb two NIR photons transferred from Yb^3+^, promoting electrons from the ground state (^4^I_15/2_) to green-emitting levels (^2^H_11/2_/^4^S_3/2_) and red-emitting levels (^4^F_9/2_). Radiative transitions back to the ground state then generate green (520-560 nm) and red (645-680 nm) emissions (Fig. 4f, left). Similarly, Yb^3+^/Tm^3+^ co-doping enables three-photon absorption processes that populate the blue-emitting ^1^G_4_ level in Tm³⁺, yielding 475 nm emission through ^1^G_4_ → ^3^H_6_ transitions. Leveraging this spectral tunability, we developed multi-color UCL nanoparticles. Uniform *β*-NaYF_4_ nanorods were synthesized with controlled Yb^3+^/Er^3+^ and Yb^3+^/Tm^3+^ doping ratios: blue (Gd/Yb/Tm: 40/18/0.5 mol%), green (Gd/Yb/Er: 40/18/2 mol%), yellow (Gd/Yb/Er: 40/58/2 mol%), and red (Gd/Yb/Er: 40/59.95/0.05 mol%) emitters (Fig. 4f, right). In our system, increasing the effective Yb/Er ratio leads to a progressive increase in the R/G emission ratio, resulting in a color transition from green to yellow and ultimately to red. A detailed mechanistic explanation of the emission color tuning in the UCNPs is provided in Note S4. To ensure uniform mixing with the SF matrix, the UCNPs were subjected to an acid-washing treatment to remove surface oleate ligands and render them water-dispersible (Fig. S12), thereby enabling homogeneous dispersion within the SF matrix^62^. Incorporation of these UCNPs into SF solutions produced flexible films with distinct blue, green, yellow, and red UCL (Fig. 4g). Comparative analyses of luminescent spectra and decay curves indicate that the SF matrix does not significantly alter the luminescent properties of the UCNPs (Fig. S17 and Note S5). This multicolor platform establishes the foundation for multi-channel information encoding and encryption using wavelength-multiplexed SF substrates.

### A soft robotic gripper carrying holographic instructions for object sorting

By modularly assembling the actuation module with the optical functional unit (LCN holography alone or integrated with UCNP-decorated SF), entirely soft robotic systems with embedded photonic intelligence can be constructed. Through the incorporation of real-time optical feedback, where extracted holographic signals are directly translated into control commands, the OISRS enables task-specific motions without reliance on external electronic device outputs or human cognition. More importantly, the encoded optical commands can only be precisely extracted with the guidance of the “instruction book” (circularly polarized laser illumination), enabling enhanced storage security. Additionally, the holographic encoding strategy presented here offers an effective approach for tamper-resistant command transmission in soft robotic systems, offering distinct advantages over conventional electronically controlled platforms.

**Fig. 5:**
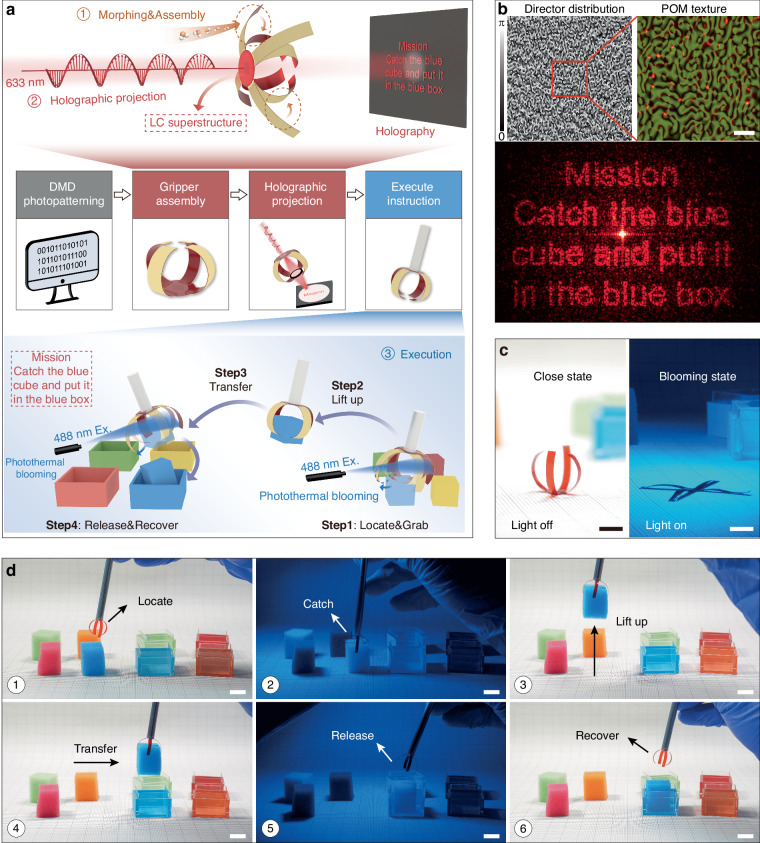
An optically interactive soft robotic gripper for object sorting. **a** Schematics of the gripper for precise on-demand grasping and sorting via holographic feedback-driven control. **b** The LC director distribution, POM texture, and holographic image corresponding to the designed mission. Scale bar: 75 μm. **c** Gripper in the closed state (left) with light off, and in the blooming state (right) under light illumination. Scale bars: 1 cm. **d** The entire process of locating, catching, transferring, and releasing using the soft gripper. Light intensity: ~ 0.25 W/cm^2^. Scale bars: 1 cm

As a proof of concept, we demonstrate an optically interactive soft gripper capable of precise, on-demand object manipulation via optical feedback-driven control (Fig. 5a). We fabricated a four-armed soft gripper, with each arm composed of an LCN/SF bilayer structure shaped via high-humidity treatment. The arms were mounted on a circular LCN film base embedded with photopatterned LC phase holograms for storing and displaying command information (Fig. 5a, top). Thanks to the LC’s superior pattern encoding capability, the resulting holographic image demonstrates high fidelity to the intended design, providing sufficient quality to support reliable command delivery in subsequent operations (Fig. 5b). Besides, the LCN/SF bilayer’s good photo-responsiveness enables the gripper to achieve a large blooming amplitude, transitioning from its initial humidity-induced closed configuration to a fully expanded state under light irradiation (Fig. 5c and Movie S4), thereby ensuring effective grasping.

The grasping task can be accurately executed under the guidance of holographically encoded command signals. The entire grasping procedure is illustrated at the bottom of Fig. 5a and physically shown in Fig. 5d and Movie S5. Firstly, the gripper was positioned above the blue cube. Upon blue light irradiation, the gripper arms bloomed and subsequently grasped the cube immediately following light cessation (Step 1, ①-②). The object was then lifted and transferred to the target location atop the blue box (Step 2-3, ③-④). Throughout this transfer, the SF films’ robust shape-fixing capability maintains grip stability, significantly simplifying gripper operation. Finally, when re-exposed to light, the gripper re-opened to release the cube, allowing it to drop into the blue box while the gripper returned to its initial configuration (Step 4, ⑤-⑥). This demonstration conclusively validates that our OISRS achieves light-controlled command transmission and task execution, paving the way for the development of fully optically intelligent soft machines.

### A walking robot integrated with hierarchically encrypted holographic instructions for maze escape

For sensitive mission operations requiring strict instruction confidentiality, a multi-level, multiplexing encryption strategy should be established to achieve advanced security protection. The execution terminal must sequentially decode each encryption layer to access the final valid instructions. To achieve this, we can integrate holographic projection, fluorescence emission, and 3D morphing technologies within soft robots, enabling covert embedding and controllable decoding of encrypted commands via multimodal signal coupling. To demonstrate this concept, we developed a maze-navigating robot incorporating hierarchically encrypted optical information.

**Fig. 6:**
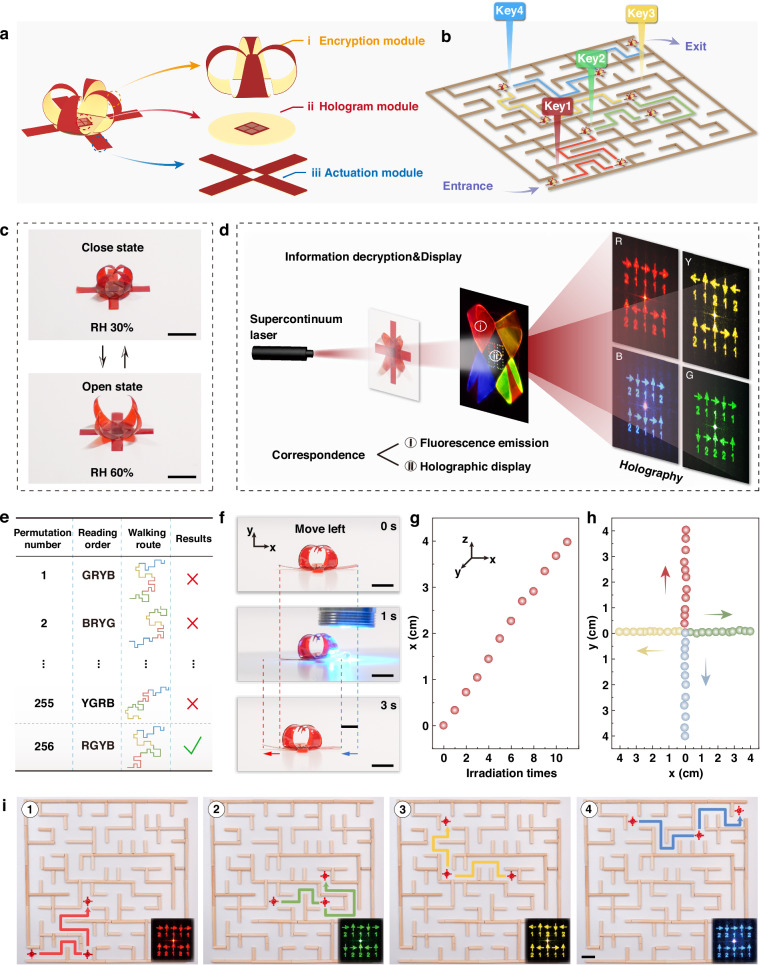
A hierarchically encrypted, optically interactive walking robot for maze escape. **a** Schematic illustration of the walking robot’s hierarchical design, including encryption, hologram, and actuation modules. **b** Schematic illustration of the maze escape process according to the four decrypted command messages. **c** Transition of the flower structure from a closed to an open state under water mist, revealing the underlying hologram module. Scale bars: 5 mm. **d** The extraction of four holographic commands by a supercontinuum laser ii), which correspond uniquely to the fluorescence emission from each of the four petals under 980 nm laser irradiation i). **e** Walking trajectories and escape results corresponding to four color-coded instructions under various reading sequences. **f** Locomotion of the walking robot driven by a 488 nm laser. Scale bars: 5 mm. **g** Lateral displacement of a leg along the x-axis as a function of the number of laser exposures. **h** Trajectories for the four movement direction scenarios with the continuous on/off operation of the laser. **i** Digital photos of the walking robot escaping the maze based on the specific path-guiding instructions (insets). Scale bars: 2 cm

This OISRS adopts a modular architecture following the “encryption-display-processing-execution” workflow. In the information encryption module, four triangular LCN/SF bilayers were engineered, shape-morphed, and assembled into a closed, flower-like structure. UCNPs, exhibiting four distinct excitation-dependent fluorescent colors, were pre-incorporated into the SF film layer of the flower petals (Fig. 6a-i). For the hologram module, an LCN-based holographic disk was developed, featuring four distinct LCN holograms, each capable of displaying unique command information (Fig. 6a-ii). Finally, for the information execution module, a soft robotic walker was designed with four splay-aligned LCN/SF legs, mimicking the morphology of a starfish, to facilitate smooth omnidirectional crawling (Fig. 6a-iii). Overall, the walking robot we fabricated has a mass of 14.2 mg, with dimensions of 1.5 cm in length, 1.5 cm in width, and 5 mm in height (in the closed state).

By progressively decrypting the four command messages encoded within the LCN holograms and acquiring the predefined secret code, the OISRS can be successfully guided to escape from the maze (Fig. 6b). Specifically, the flower structure is initially in a closed bud state and blooms upon exposure to water mist (Fig. 6c, 1st encryption layer). Once opened, the four holographic commands encoded within the LCN superstructures on the disk can be projected using a circularly polarized supercontinuum laser (Fig. 6d, 2nd encryption layer). Subsequently, the fluorescence emission from each of the four petals, corresponding uniquely to one of the four holographic projection patterns, can be extracted under 980 nm laser irradiation (3rd encryption layer). Finally, a security key (4th encryption layer), derived from the fluorescent color sequence (B, G, Y, R), decodes a unique and validated set of path-guiding instructions. With four colors, 256 (4⁴ = 256) possible permutations exist, and we specified the sequence “RGYB” as the secret code (Fig. 6e). Thanks to the high holographic efficiency of the LC superstructures across multiple wavelengths and the broad-spectrum, high transmittance of the SF film (Fig. S13), the holographic commands can be stably displayed and efficiently transmitted over a wide spectral range (Fig. S14), thereby enhancing the environmental adaptability of the system. Altogether, this OISRS establishes a quadruple-layer encryption architecture that substantially elevates command information security, preventing unauthorized access attempts. A more detailed explanation regarding the functions of the four petals and the entire encryption process is shown in Note S6.

Based on the validated command signals, we subsequently investigated the OISRS’s information execution behaviors. To this end, we first examined the light-driven actuation performance of this soft robotic system. When an LCN leg was irradiated near one end, it exhibited downward bending, thereby generating asymmetric elastic energy accumulation within the structure (Fig. S15). Upon removing the light source, the release of stored elastic energy induced macroscopic displacement of the entire system toward the other end. For instance, to achieve a leftward movement, the right-side LCN leg was selectively activated, leading to a lateral displacement of approximately 3.5 mm (Fig. 6f). Under periodic light stimulation, the OISRS exhibits linearly cumulative displacement with increasing exposure cycles (Fig. 6g), demonstrating the stability and continuity of its actuation performance. Four distinct trajectories recorded on the x–y plane further demonstrate the stable translocation in all directions (Fig. 6h). Under different light intensities, the robot exhibits varying locomotion speeds, with a maximum speed of approximately 1.33 mm/s under our experimental conditions (Fig. S16). Through the coordinated operation of these actuation components, we actuated the walking robot to navigate and successfully escape the maze, thereby fulfilling the designed objective (Fig. 6i and Movie S6).

## Discussion

In summary, we have successfully established LC holography technology as a promising approach for enhancing soft robots with non-electronic, high-function-density, and secure information storage and processing capabilities. The unique combination of a programmable LC polymer and a versatile biopolymer material not only creates a unified platform that simultaneously achieves multi-degree-of-freedom actuation and high-capacity optical information multiplexing through their functional interplay but also opens new opportunities for biohybrid robotic systems operating at the biology-technology interface by leveraging their inherent biocompatibility^63–67^. The ability to precisely control the phase hologram at the microscale opens new possibilities for optically interactive microrobotic technologies. Moreover, the advancement of LC computational holography, characterized by high resolution, real-time dynamics, and multi-dimensional control, would significantly enhance the optical interaction intelligence of soft robotic systems.

Furthermore, by integrating real-time optical sensory feedback, this OISRS holds promise for breakthroughs in autonomy, precision, and adaptability, bridging the gap between artificial systems and biological intelligence. Specifically, the integration of actuation and information processing within a biocompatible soft-matter architecture may enable a distinctive application scenario: cooperative optical inter-robot microsurgery. In this envisioned ecosystem, external doctor robots (macrobots, not demonstrated in our work) interact with implanted soft micro-agents (our OISRS) to perform precision therapies in environments where electronic devices are impractical (e.g., magnetic resonance imaging-guided procedures). The workflow relies on a unique “optical handshake”: (i) Upon injection, the micro-agent utilizes humidity-responsive morphing to anchor within the tissue; (ii) The macrobot verifies the agent’s identity via UCNP-based fluorescence encryption, preventing medical errors; (iii) Following holographic status feedback, the macrobot triggers light-driven actuations for targeted drug release or grasping. Nevertheless, we acknowledge that bridging the gap between this vision and reality presents challenges. While the demonstrated near-infrared upconversion offers better penetration, the actuation wavelengths for LCNs need to be further optimized towards the biological transparency windows (NIR-I or NIR-II) to minimize tissue damage and signal attenuation. Alternatively, this optical interaction strategy may be implemented via minimally invasive interfaces, such as optical fiber catheters or endoscopes, which can deliver excitation light and collect holographic/fluorescent signals directly at the target site. With the advent of the era of intelligent robotics, future iterations are expected to address these restricts. This work provides a transformative paradigm for the development of next-generation all-optical intelligent, entirely soft robotics, merging adaptive mechanical performance with sophisticated optical information processing.

## Materials and methods

### Materials

RM257 and RM105 (LC monomer) were purchased from HCCH, China. DR1A was purchased from Sigma-Aldrich. The photoinitiator Irgacure 651 was purchased from KingChem, China. 5CB was purchased from Nanjing Leyao Technology Co., Ltd. SD1 was purchased from Dai-Nippon Ink and Chemicals, Japan. DMOAP (60 wt% in methanol) was purchased from Aladdin. *Bombyx mori* silkworm cocoons were purchased from local farmers in Nantong. Sodium carbonate (Na_2_CO_3_, 99.5%) was obtained from Sinopharm Chemical Reagent Co., Ltd. Lithium bromide (LiBr, 99%) was purchased from Macklin Biochemical Co., Ltd. Trichloro (^1^H,^1^H,^2^H,^2^H-perfluorooctyl) silane (97%) was purchased from Xushuo Biotechnology Co., Ltd. YCl_3_·6H_2_O (99.99% trace metals basis), YbCl_3_·6H_2_O (99.99% trace metals basis), ErCl_3_·6H_2_O (99.99% trace metals basis), TmCl_3_·6H_2_O (99.99% trace metals basis) and oleic acid (90%) were purchased from Sigma-Aldrich. NaOH (99.9% metals basis), NH_4_F ( ≥ 99.99% metals basis) were purchased from Aladdin. Ethanol ( ≥ 99.8%) and HCl (36.0 ~ 38.0%) were purchased from Sinopharm Chemical Reagent Co., Ltd.

### Preparation of splay cells

To induce planar molecular alignment, pre-cleaned glass substrates were spin-coated with photoalignment agent SD1. For unidirectional photoalignment, the substrates were irradiated with linearly polarized light (405 nm, Thorlabs M405P1) at an intensity of 3.86 mW cm^-2^ for 15 min. The in-plane orientation of the liquid crystal director was defined by the polarization direction of the incident light. For homeotropic anchoring, pre-cleaned glass substrates were dip-coated in a 3% v/v aqueous solution of DMOAP for 30 min, followed by baking at 100 °C for 10 min. Splay cells were assembled by pairing one planar-aligned and one homeotropic-treated substrate, with the cell gap defined by 50 μm-thick spacers.

### Fabrication of splay-structured LCN films

The LCN precursor mixture used in all samples comprised 37 wt% RM257, 60 wt% RM105, 2 wt% DR1A, and 1 wt% Irgacure 651. To this mixture, 5CB was added at 20 wt% relative to the total mass of the LCN components. All mixtures were heated to 100 °C to reach the isotropic phase, stirred for 1 h, and subsequently injected into splay-aligned cells. The filled cells were cooled to 45 °C at a controlled rate of ∼1 °C min^−1^, followed by UV irradiation at 365 nm (Herolab, ∼6 mW·cm^−2^) for 1 h to initiate crosslinking. After polymerization, the cell was opened with a blade to obtain a freestanding LCN film.

### Preparation of LCN films with embedded phase holograms

Two SD1-coated glass substrates were assembled to form a cell, with the thickness controlled by 10 μm spacers. For patterned photoalignment, the empty cell was positioned at the image plane of a digital micro-mirror device-based microlithography system to record the patterns of LC director distribution via a multistep, partly overlapping exposure process with synchronous polarization control. Subsequently, LCN films embedded with phase holograms were fabricated following the same procedure described above.

### Fabrication of SF solution and films

The regenerated SF solution was extracted from the cocoons of the *Bombyx mori* silkworm using established protocols^68^. Briefly, the silk cocoons cut into small pieces were boiled in a solution of Na_2_CO_3_ (0.02 M) for 30 min and thoroughly washed with deionized water to remove the sericin. After drying under ambient air for 2 days, the degummed silk fibers were dissolved in a LiBr solution (9.3 M) at 60 °C for 4 h. Following this, the dissolved SF was dialyzed in deionized water with dialysis bags (molecular-weight cutoff: 3500) for 3 days. The SF solution, with a concentration of ~6–7 wt%, was obtained by centrifuging at 11,000 rpm for 20 min, followed by two rounds of filtration using dust-free paper. An amorphous SF cast film was prepared by depositing a certain volume of SF solution (volume-adjusted for target thickness) onto a silicon wafer treated with trichloro(^1^H,^1^H,^2^H,^2^H-perfluorooctyl) silane, followed by ambient drying overnight.

### Synthesis of the ligand-free lanthanide-doped NaYF_4_ nanorods

In a typical procedure for synthesizing lanthanide-doped NaYF_4_ nanorods, 1.5 mL of an aqueous NaOH solution (0.3 g NaOH) was mixed with 5 mL of ethanol and 5 mL of oleic acid under continuous stirring. Subsequently, 2 mL of RECl_3_ solution (0.2 M; RE = Y, Yb, Er, Tm, and Gd) and 1 mL of NH_4_F solution (2 M) were selectively added. The resulting mixture was transferred into a 20 mL Teflon-lined autoclave and heated at 200 °C for 2 h. The obtained nanorods were collected by centrifugation and washed multiple times with water and ethanol. The compositions of the nanorods emitting blue, green, yellow, and red light were NaYF_4_:Gd/Yb/Tm (40/18/0.5 mol%), NaYF_4_:Gd/Yb/Er (40/18/2 mol%), NaYF_4_:Gd/Yb/Er (40/58/2 mol%), and NaYF_4_:Gd/Yb/Er (40/59.95/0.05 mol%), respectively. For ligand removal, approximately 10 mg of oleic acid-capped nanorods were dispersed in 0.75 mL of ethanol via sonication and mixed with 0.75 mL of 1 M aqueous HCl. The mixture was sonicated for 1 minute, followed by centrifugation at 16,000 rpm for 15 min. The acid treatment was repeated twice to ensure the complete removal of ligands. The purified nanorods were further washed with ethanol and redispersed in water for subsequent use. Notably, the concentration of HCl can be adjusted based on the type of nanomaterials.

### Characterization

The photographs and videos of samples were recorded by a digital camera (EOS 850D, Canon, Japan). The POM textures were taken by a polarized optical microscope (DM2700P, Leica, Germany). A supercontinuum laser (SuperK EVO, NKT Photonics, Denmark) combined with a multichannel acousto-optic tunable filter (SuperK SELECT, NKT Photonics, Denmark) was adopted as the laser source to illuminate the LCN hologram, and the holographic images were recorded by a CCD camera (E3ISPM12000KPB, Guangzhou Weiyu Optical Instrument, China). The morphologies of the bilayer structure were observed with a field emission scanning electron microscope (ULTRA 55, Zeiss, Germany) at a voltage of 5 kV and a current of 10 μA. The morphologies of NaYF_4_ nanorods were observed using a transmission electron microscopy instrument (JEOL/JEM-1400FLASH, Japan). 488 nm laser (MW-GX-488, Changchun Laser Optoelectronics Technology Co., Ltd., China) was used for light-driven actuation. The photothermal-induced temperature variation of the samples was recorded by an infrared camera (628 C, Fotric Inc., China). A 980 nm laser (MDL-XF-980, Changchun Laser Optoelectronics Technology Co., Ltd., China) was used for upconversion luminescence. The water vapor was generated using an enclosed thermostatic water bath device (NK-420, Nuoji Instrument, China), where the temperature and the amount of water vapor can be precisely set and maintained. A humidifier (buresBE-J001, Bures, China) was used to generate water mist. The bending angles and curvatures of bilayer strips were analyzed by ImageJ.

## Supplementary information

Supplementary Information

Movie S1

Movie S2

Movie S3

Movie S4

Movie S5

Movie S6

## Data Availability

All data are available in the main text or the supplementary information.

## References

[1] Rus, D. & Tolley, M. T. Design, fabrication and control of soft robots. *Nature* **521**, 467–475 (2015). 26017446 10.1038/nature14543

[2] Rich, S. I., Wood, R. J. & Majidi, C. Untethered soft robotics. *Nat. Electron.* **1**, 102–112 (2018).

[3] Rothemund, P. et al. Shaping the future of robotics through materials innovation. *Nat. Mater.* **20**, 1582–1587 (2021). 34815572 10.1038/s41563-021-01158-1

[4] Bang, J. et al. Bioinspired electronics for intelligent soft robots. *Nat. Rev. Electr. Eng.* **1**, 597–613 (2024).

[5] Cianchetti, M. et al. Biomedical applications of soft robotics. *Nat. Rev. Mater.* **3**, 143–153 (2018).

[6] Li, M. et al. Soft actuators for real-world applications. *Nat. Rev. Mater.* **7**, 235–249 (2022). 35474944 10.1038/s41578-021-00389-7 PMC7612659

[7] Li, G. R. et al. Bioinspired soft robots for deep-sea exploration. *Nat. Commun.* **14**, 7097 (2023). 37925504 10.1038/s41467-023-42882-3 PMC10625581

[8] Zhang, Y. C. et al. Progress, challenges, and prospects of soft robotics for space applications. *Adv. Intell. Syst.* **5**, 2200071 (2023).

[9] Wang, S. et al. Asymmetric elastoplasticity of stacked graphene assembly actualizes programmable untethered soft robotics. *Nat. Commun.* **11**, 4359 (2020). 32868779 10.1038/s41467-020-18214-0 PMC7459344

[10] Dong, Y. et al. Untethered small-scale magnetic soft robot with programmable magnetization and integrated multifunctional modules. *Sci. Adv.* **8**, eabn8932 (2022). 35731876 10.1126/sciadv.abn8932 PMC9217092

[11] Sun, M. M. et al. Bioinspired self-assembled colloidal collectives drifting in three dimensions underwater. *Sci. Adv.* **9**, eadj4201 (2023). 37948530 10.1126/sciadv.adj4201 PMC10637755

[12] Sinatra, N. R. et al. Ultragentle manipulation of delicate structures using a soft robotic gripper. *Sci. Robot.* **4**, eaax5425 (2019). 33137785 10.1126/scirobotics.aax5425

[13] Zhang, N. B. et al. Soft robotic hand with tactile palm-finger coordination. *Nat. Commun.* **16**, 2395 (2025). 40064944 10.1038/s41467-025-57741-6 PMC11894155

[14] Sankar, S. et al. A natural biomimetic prosthetic hand with neuromorphic tactile sensing for precise and compliant grasping. *Sci. Adv.* **11**, eadr9300 (2025). 40043132 10.1126/sciadv.adr9300 PMC11881920

[15] Liu, X. R. et al. Magnetic soft microfiberbots for robotic embolization. *Sci. Robot.* **9**, eadh2479 (2024). 38381840 10.1126/scirobotics.adh2479

[16] Wang, B. et al. Magnetically driven biohybrid blood hydrogel fibres for personalized intracranial tumour therapy under fluoroscopic tracking. *Nat. Biomed. Eng.* **9**, 1471–1485 (2025). 40312457 10.1038/s41551-025-01382-z

[17] Jiang, J. W. et al. NIR-II fluorescent thermophoretic nanomotors for superficial tumor photothermal therapy. *Adv. Mater.* **37**, 2417440 (2025). 39895191 10.1002/adma.202417440 PMC11899490

[18] Liang, J. M. et al. Electrostatic footpads enable agile insect-scale soft robots with trajectory control. *Sci. Robot.* **6**, eabe7906 (2021). 34193563 10.1126/scirobotics.abe7906

[19] Fan, X. J. et al. Scale-reconfigurable miniature ferrofluidic robots for negotiating sharply variable spaces. *Sci. Adv.* **8**, eabq1677 (2022). 36112686 10.1126/sciadv.abq1677 PMC9481141

[20] Yang, X. et al. Milli-scale cellular robots that can reconfigure morphologies and behaviors simultaneously. *Nat. Commun.* **13**, 4156 (2022). 35851279 10.1038/s41467-022-31913-0 PMC9293897

[21] Liu, W. B. et al. Touchless interactive teaching of soft robots through flexible bimodal sensory interfaces. *Nat. Commun.* **13**, 5030 (2022). 36028481 10.1038/s41467-022-32702-5 PMC9412806

[22] Lei, B. & Chen, L. J. Light-driven bionic liquid crystal elastomers soft actuators. *Chin. J. Liq. Cryst. Disp.* **39**, 707–724 (2024).

[23] Lei, J. G. et al. Design of holographic reproduction images based on liquid crystal spatial light modulator. *Chin. Opt.* **18**, 771–783 (2025).

[24] Zhang, J. R. et al. Multidimensional multiplexing geometric phase metaholography. *Light.: Adv. Manuf.* **6**, 474–485 (2025).

[25] Hsu, C. G., Xu, Z. D. & Wang, X. G. Holographic recording and hierarchical surface patterning on periodic submicrometer pillar arrays of azo molecular glass via polarized light irradiation. *Adv. Funct. Mater.* **28**, 1802506 (2018).

[26] Jiang, C. et al. All-protein-based rewritable and reprogrammable multifunctional optical imaging platforms via multi-strategy imprinting and multimode 3D morphing. *Matter* **7**, 1591–1611 (2024).

[27] Wang, X. J. et al. Exploiting universal nonlocal dispersion in optically active materials for spectro-polarimetric computational imaging. *eLight* **4**, 22 (2024).

[28] Yao, M. et al. High-security plastic with integrated holographic and phosphorescent images. *Adv. Mater.* **37**, 2414894 (2025). 10.1002/adma.202414894 39972958

[29] Wang, Z. Y. et al. Cascaded liquid crystal holography for optical encryption. *Chin. Opt. Lett.* **21**, 120003 (2023).

[30] Wang, Z. Y. et al. Vectorial liquid-crystal holography. *eLight* **4**, 5 (2024).

[31] Tang, D. L. et al. Simultaneous surface display and holography enabled by flat liquid crystal elements. *Laser Photonics Rev.* **16**, 2100491 (2022).

[32] Liu, S. J. et al. Bi-chiral nanostructures featuring dynamic optical rotatory dispersion for polychromatic light multiplexing. *Adv. Mater.* **35**, 2301714 (2023). 10.1002/adma.202301714 37158735

[33] Xu, X. et al. Reprogrammable vector optical field meets planar liquid crystal elements for enhanced security in holography. *Laser Photonics Rev.* **19**, 2401962 (2025).

[34] White, T. J. & Broer, D. J. Programmable and adaptive mechanics with liquid crystal polymer networks and elastomers. *Nat. Mater.* **14**, 1087–1098 (2015). 26490216 10.1038/nmat4433

[35] Shahsavan, H. et al. Bioinspired underwater locomotion of light-driven liquid crystal gels. *Proc. Natl. Acad. Sci. USA* **117**, 5125–5133 (2020). 32094173 10.1073/pnas.1917952117 PMC7071923

[36] Li, Y., Liu, Y. J. & Luo, D. Polarization dependent light-driven liquid crystal elastomer actuators based on photothermal effect. *Adv. Opt. Mater.* **9**, 2001861 (2021).

[37] Zhuang, X. L. et al. Active terahertz beam steering based on mechanical deformation of liquid crystal elastomer metasurface. *Light Sci. Appl.* **12**, 14 (2023). 36596761 10.1038/s41377-022-01046-6 PMC9810742

[38] Wang, Y. S., Li, M. & Wang, Y. Silk: a versatile biomaterial for advanced optics and *photonics*. *Chin. Opt. Lett.* **18**, 080004 (2020).

[39] Podbevšek, D. et al. The role of water mobility on water-responsive actuation of silk. *Nat. Commun.* **15**, 8287 (2024). 39333569 10.1038/s41467-024-52715-6 PMC11436739

[40] Wang, Y. L., Feng, X. & Chen, X. D. Autonomous bioelectronic devices based on silk fibroin. *Adv. Mater.* **37**, 2500073 (2025). 10.1002/adma.202500073 40123251

[41] Wang, Y. et al. Light-activated shape morphing and light-tracking materials using biopolymer-based programmable photonic nanostructures. *Nat. Commun.* **12**, 1651 (2021). 33712607 10.1038/s41467-021-21764-6 PMC7955034

[42] Xiao, J. L. et al. Reprogrammable multi-responsiveness of regenerated silk for versatile soft actuators. *Adv. Funct. Mater.* **34**, 2316301 (2024).

[43] Chen, G. K. et al. A triple-shape memory material via thermal responsive behavior of liquid crystalline network incorporating main-chain/side-chain LC units. *Macromol. Chem. Phys.* **220**, 1900059 (2019).

[44] Jiang, Z. C. et al. Selective decrosslinking in liquid crystal polymer actuators for optical reconfiguration of origami and light-fueled locomotion. *Angew. Chem.* **131**, 5386–5391 (2019). 10.1002/anie.201900470 30816599

[45] Jiang, J. et al. Porous liquid-crystalline networks with hydrogel-like actuation and reconfigurable function. *Angew. Chem.* **134**, e202116689 (2022). 10.1002/anie.202116689 34970834

[46] Fu, C. J., Porter, D. & Shao, Z. Z. Moisture effects on *Antheraea pernyi* silk’s mechanical property. *Macromolecules* **42**, 7877–7880 (2009).

[47] Ma, L. L. et al. Programmable self-propelling actuators enabled by a dynamic helical medium. *Sci. Adv.* **7**, eabh3505 (2021). 34362740 10.1126/sciadv.abh3505 PMC8346214

[48] Zheng, Z. G. et al. Digital photoprogramming of liquid-crystal superstructures featuring intrinsic chiral photoswitches. *Nat. Photonics* **16**, 226–234 (2022).

[49] Zheng, R. et al. Stimuli-responsive active materials for dynamic control of light field. *Responsive Mater.* **1**, e20230017 (2023).

[50] Xu, Y. Y. et al. Modulating the macroscopic anisotropy of liquid crystalline polymers by polarized light. *Responsive Mater.* **2**, e20240020 (2024).

[51] Li, S. et al. When quantum dots meet blue phase liquid crystal elastomers: visualized full-color and mechanically-switchable circularly polarized luminescence. *Light Sci. Appl.* **13**, 140 (2024). 38876989 10.1038/s41377-024-01479-1 PMC11178798

[52] Ren, Y. R. et al. Programmable polarization and structural color in a stretchable luminescent chiral liquid crystal elastomer. *Responsive Mater.* **3**, e70030 (2025).

[53] Ma, L. L. et al. Self-assembled liquid crystal architectures for soft matter photonics. *Light Sci. Appl.* **11**, 270 (2022). 36100592 10.1038/s41377-022-00930-5 PMC9470592

[54] Zheng, R. et al. Autonomous self-sustained liquid crystal actuators enabling active photonic applications. *Adv. Funct. Mater.* **33**, 2301142 (2023).

[55] Pan, J. T. et al. Nonlinear geometric phase coded ferroelectric nematic fluids for nonlinear soft-matter photonics. *Nat. Commun.* **15**, 8732 (2024). 39384797 10.1038/s41467-024-53040-8 PMC11464912

[56] Li, S. L. et al. Geometric phase-encoded stimuli-responsive cholesteric liquid crystals for visualizing real-time remote monitoring: humidity sensing as a proof of concept. *Light Sci. Appl.* **13**, 27 (2024). 38263398 10.1038/s41377-023-01360-7 PMC10805905

[57] Wu, K. H. et al. Light-regulated soliton dynamics in liquid crystals. *Nat. Commun.* **15**, 7217 (2024). 39174533 10.1038/s41467-024-51383-w PMC11341711

[58] Ma, L. L. et al. Soft matter photonics: interplay of soft matter and light. *ACS Nano* **19**, 11501–11516 (2025). 40111282 10.1021/acsnano.5c02465

[59] Zheng, B. Z. et al. Rare-earth doping in nanostructured inorganic materials. *Chem. Rev.* **122**, 5519–5603 (2022). 34989556 10.1021/acs.chemrev.1c00644

[60] Cheng, X. W. et al. Recent development in sensitizers for lanthanide-doped upconversion luminescence. *Chem. Rev.* **122**, 15998–16050 (2022). 36194772 10.1021/acs.chemrev.1c00772

[61] Wei, Y. et al. Frenkel defect-modulated anti-thermal quenching luminescence in lanthanide-doped Sc_2_(WO_4_)_3_. *Angew. Chem. Int. Ed.* **62**, e202303482 (2023). 10.1002/anie.202303482 37129053

[62] Yuan, Z. et al. Paving metal–organic frameworks with upconversion nanoparticles via self-assembly. *J. Am. Chem. Soc.* **140**, 15507–15515 (2018). 30350963 10.1021/jacs.8b10122

[63] Perju, E., Paslaru, E. & Marin, L. Polymer-dispersed liquid crystal composites for bio-applications: thermotropic, surface and optical properties. *Liq. Cryst.* **42**, 370–382 (2015).

[64] Martella, D. et al. Liquid crystalline networks toward regenerative medicine and tissue repair. *Small* **13**, 1702677 (2017). 10.1002/smll.201702677 29045016

[65] Wang, Y. S. et al. Silk-protein-based gradient hydrogels with multimode reprogrammable shape changes for biointegrated devices. *Proc. Natl. Acad. Sci. USA* **120**, e2305704120 (2023). 37549277 10.1073/pnas.2305704120 PMC10434304

[66] Wang, X. Y., Yang, T. & Li, Q. Micro- and nanorobots from magnetic particles: fabrication, control, and applications. *Responsive Mater.* **2**, e20240027 (2024).

[67] Meng, X. Y., Tang, Y. Q. & Li, Q. Micro- and nanomotors in biomedical applications. *Responsive Mater.* **3**, e20250021 (2025).

[68] Rockwood, D. N. et al. Materials fabrication from *Bombyx mori* silk fibroin. *Nat. Protoc.* **6**, 1612–1631 (2011). 21959241 10.1038/nprot.2011.379 PMC3808976

