# Supplementary material for: All-in-one optically interactive soft robots with embedded liquid crystal holography
Source: Light Sci Appl. 2026 May 6;15:219. doi: 10.1038/s41377-026-02287-5 (PMC13144626; doi:10.1038/s41377-026-02287-5)
Supplement: Supplementary file 1 — Supplementary Information [file 41377_2026_2287_MOESM1_ESM.pdf]

Supplementary Information for

All-in-one optically interactive soft robots with embedded liquid crystal holography

Zi-Chen Zhang[1,#], Yang Wei[1,#], Ze-Yu Wang[1,#], Ying-Hao Fu[1], Ren Zheng[1], Ning Wang[1], Yu Wang[1]*, Ling-Ling Ma[1]*, Yan-Qing Lu[1]*

[1]National Laboratory of Solid State Microstructures, Key Laboratory of Intelligent Optical Sensing and Manipulation, College of Engineering and Applied Sciences, and Collaborative Innovation Center of Advanced Microstructures, Nanjing University, Nanjing 210023, China.

[#]These authors contributed equally to this work.

*Corresponding author.

E-mail: yuwang87@nju.edu.cn, malingling@nju.edu.cn, yqlu@nju.edu.cn

Supplementary Figures

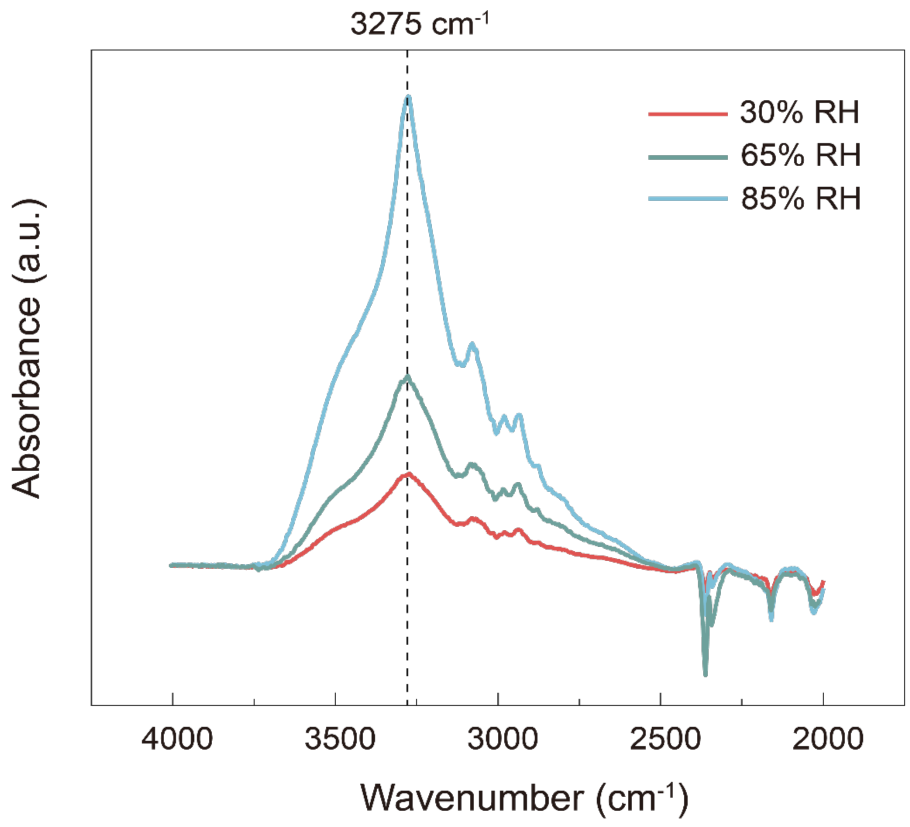

Fig. S1 FTIR spectrum of SF film under 30%, 65% and 85% RH conditions. The pronounced increase in the absorption peak at 3275 cm^{-1} (O–H stretching vibration) in the FTIR spectrum indicates substantial water absorption.

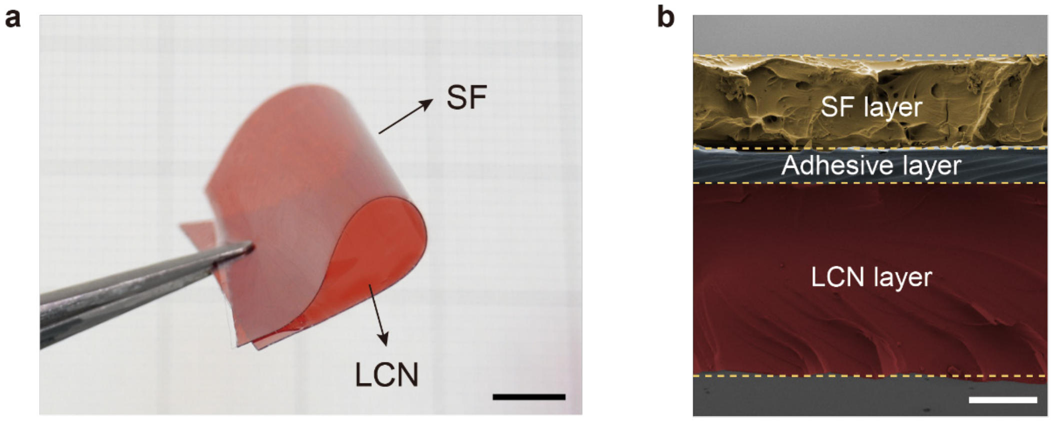

Fig. S2 The formation of a flexible and steady LCN/SF bilayer. (a) Digital photo showing the flexibility of the LCN/SF bilayer actuator. Scale bar: 5 mm. (b) Cross-sectional SEM image of the composite film, showing the compactly contacted interface between different layers. Scale bar: 20 µm.

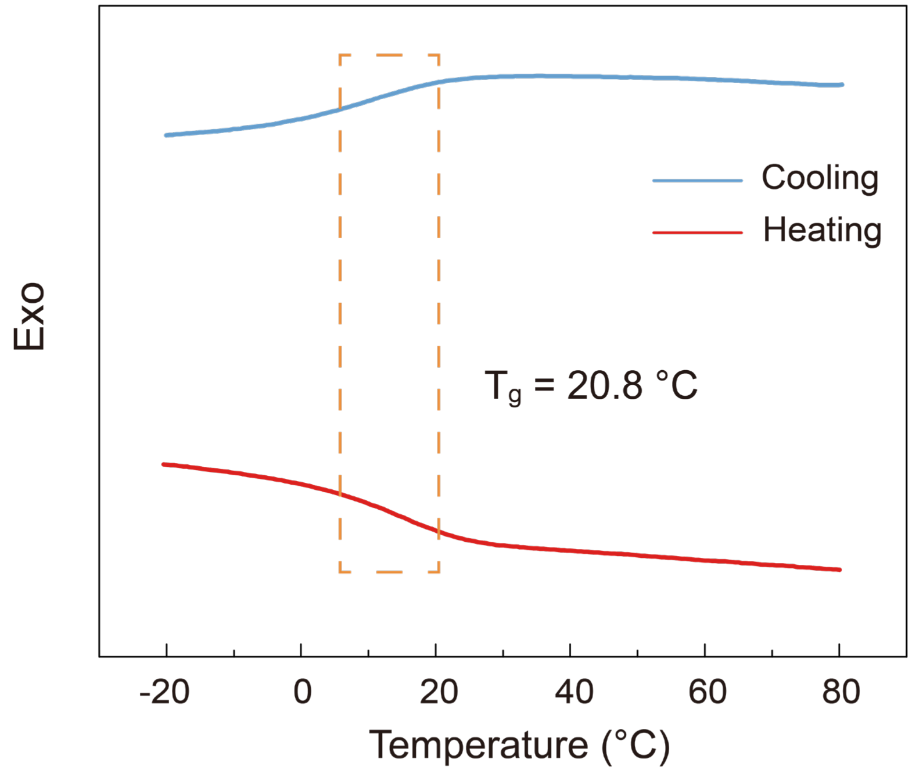

Fig. S3 Differential scanning calorimetry traces of the LCN film at heating and cooling rates of 10 °C min^{-1}. The results indicate a glass transition temperature of ~21 °C.

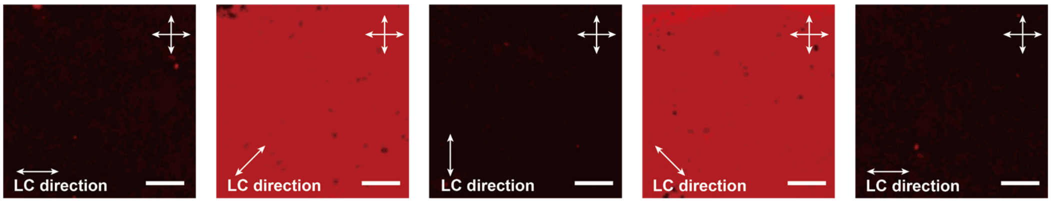

Fig. S4 Polarized optical microscope images of the LCN film captured from the PA surface under the crossed polarizers. Upon rotating the sample, the darkest and brightest states were observed when the liquid crystal molecules were oriented at 0°/90° and 45° relative to the orthogonal polarizers, respectively, indicating a highly anisotropic in-plane molecular alignment. Scale bars: 100 μm.

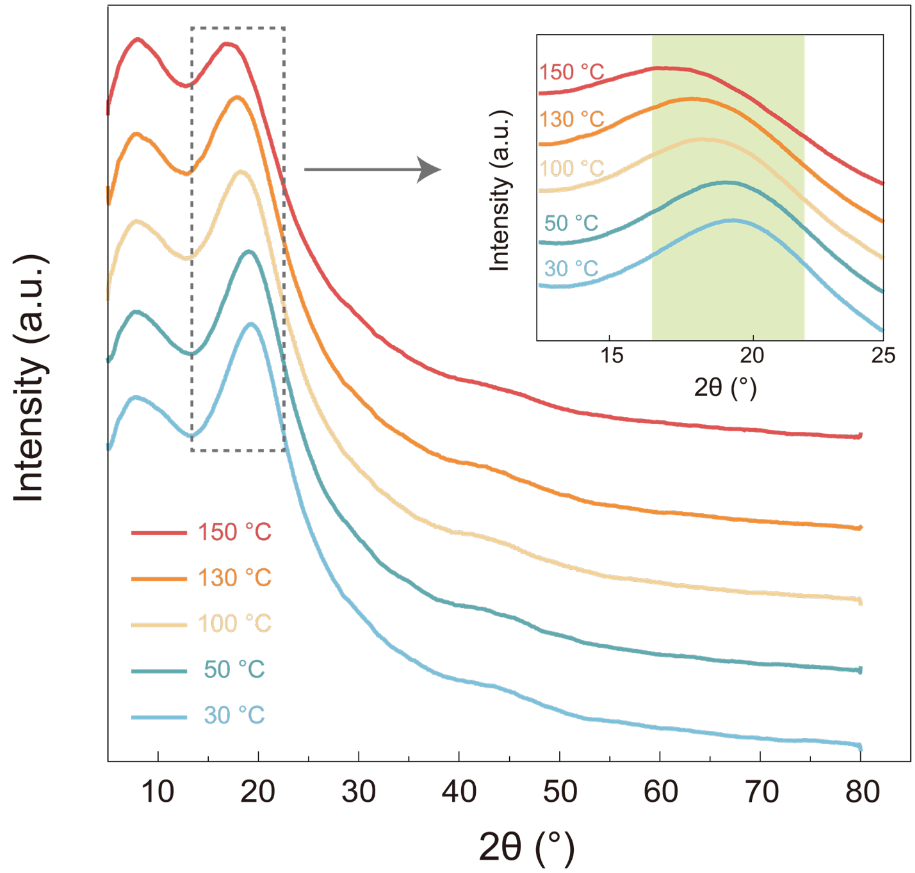

Fig. S5 XRD results of LCN at different temperature points. The diffraction intensity within the range of 18-21° decreases slightly at higher temperatures, which is attributed to enhanced atomic thermal vibration (due to the Debye-Waller effect), resulting in a reduction in peak intensity.

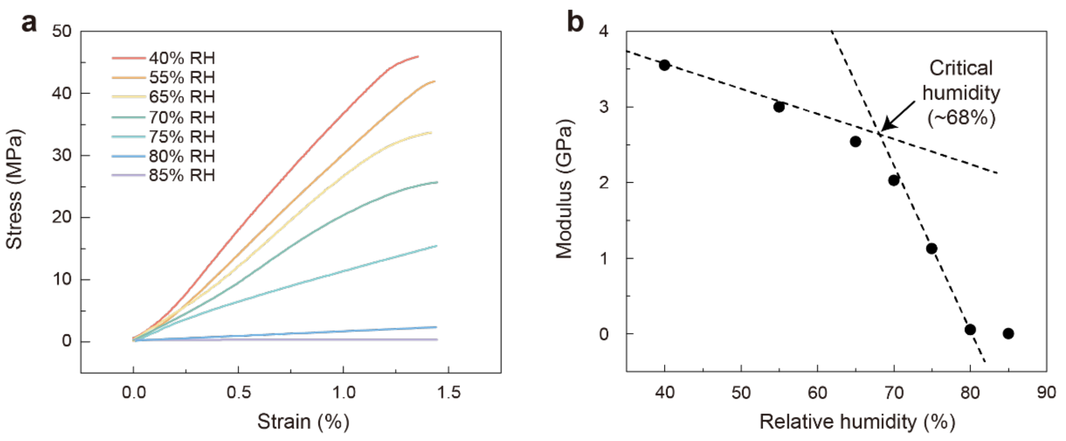

Fig. S6 Evaluation of glass transition of SF film. (a) Stress-strain curves of SF films under different RH. (b) Observation of the critical humidity in the modulus-RH curve. The results reveal a sharp modulus decrease of ~68% within this range, confirming that the glass transition occurred between 65% and 70% RH.

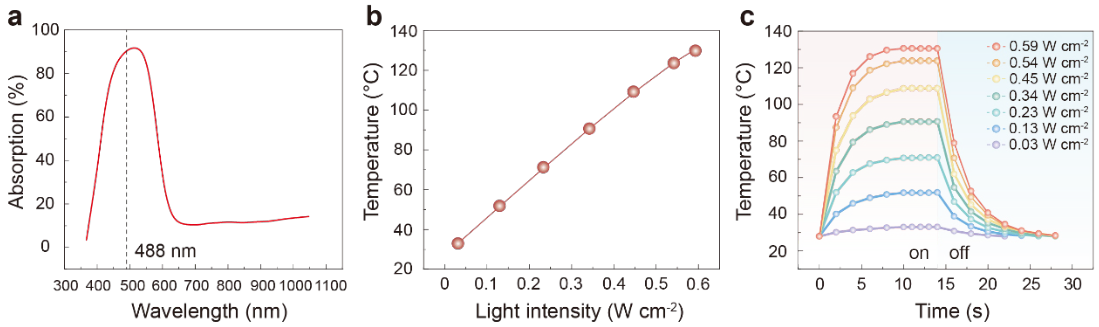

Fig. S7 Photothermal performance of the LCN/SF bilayer. (a) Absorption spectra of the dye DR1A. (b) The temperature variation of the bilayer actuator under different light intensities. (c) The temperature variation of the bilayer actuator as a function of laser illumination time under a range of light intensities.

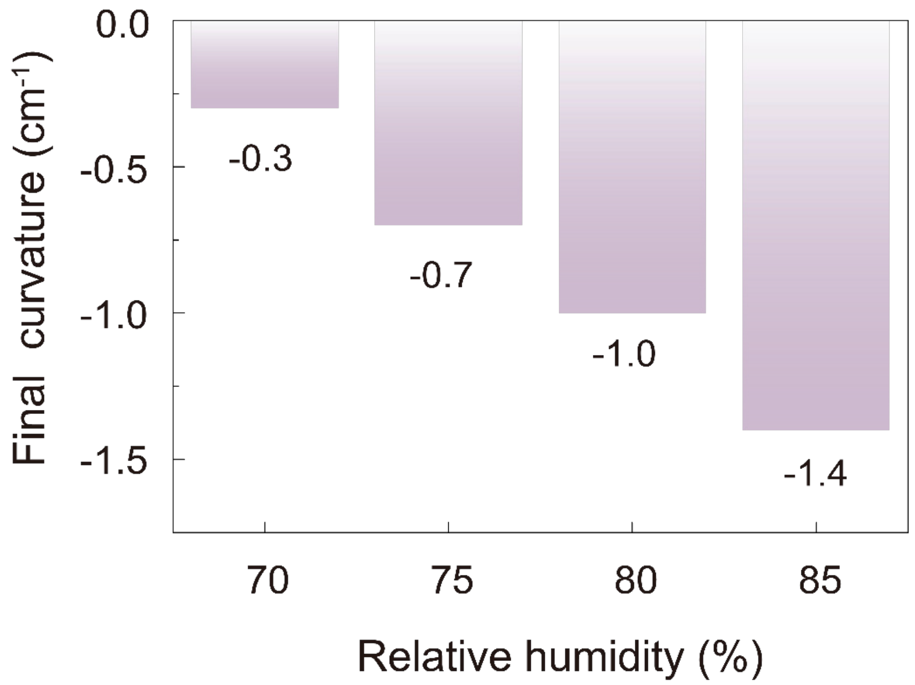

Fig. S8 Reverse bending curvature after treatments at 70%, 75%, and 80% RH and back to 30% RH.

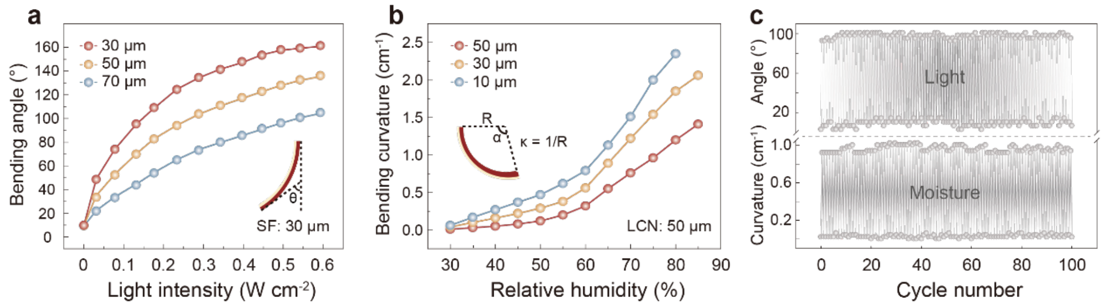

Fig S9 Actuation performance of the LCN/SF bilayer. (a) Dependence of the maximum bending angle of LCN/SF bilayers with different LCN layer thicknesses on different light intensities. The inset indicates the definition of the bending angle (θ). (b) Dependence of the maximum bending curvature of LCN/SF bilayers with different SF layer thicknesses on different RH. The inset indicates the definition of bending curvature (κ). The peak deformation curvature was characterized at relative humidity levels above 65% RH. (c) Dependence of the bending angle and curvature of the bilayer actuator on the cycle number under the stimulation of light (0.23 W/cm^2) and moisture (65%), respectively.

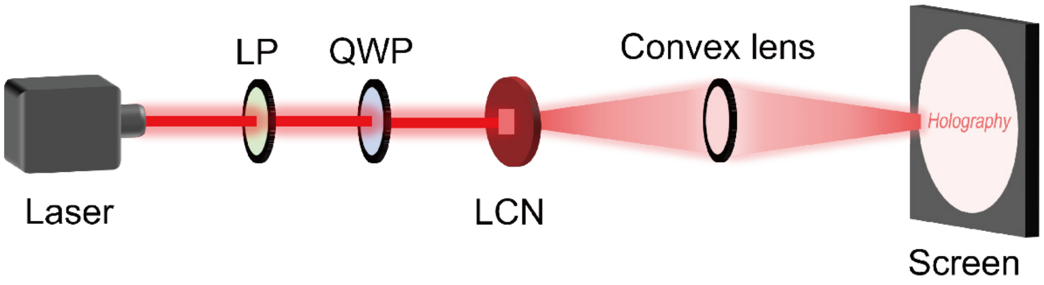

Fig. S10 Experimental setup for LCN holography extraction. Circularly polarized laser irradiation enables high-fidelity extraction of holographic information.

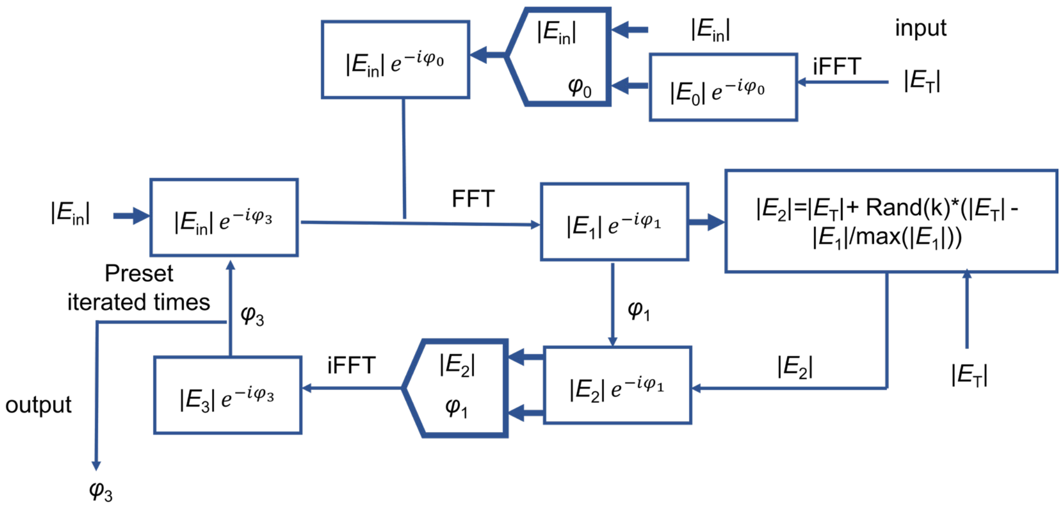

Fig. S11 Flow chart of the Gerchberg-Saxton algorithm for LCN holography.

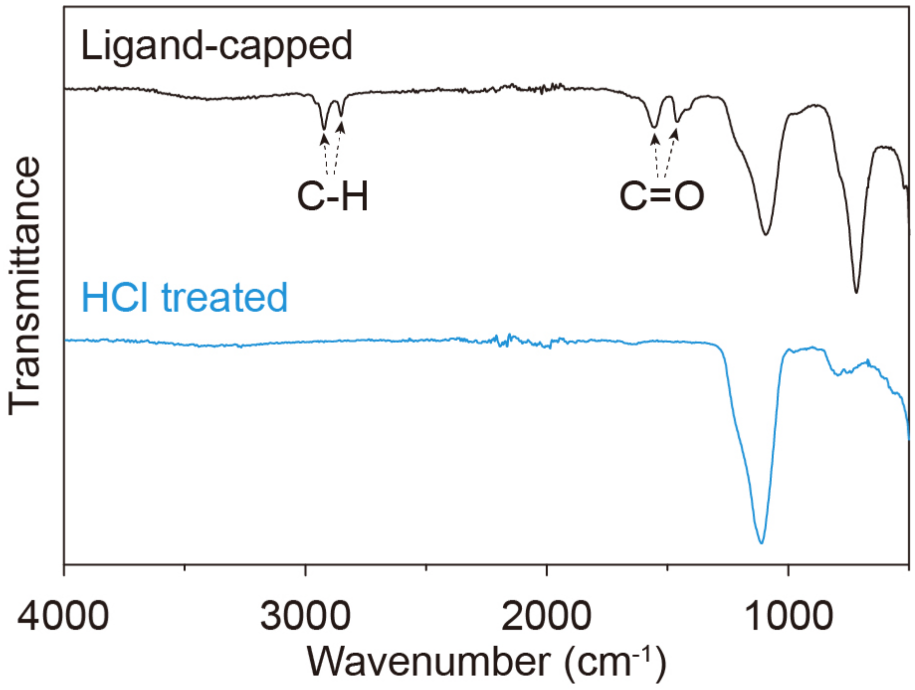

Fig. S12 FTIR spectra of ligand-capped UCNPs and HCl treated UCNPs. Chemically, HCl protonates the oleate species on the nanoparticle surface and breaks the coordination between the surface rare-earth sites and the carboxylate group. The coordinated oleate (-COO^-) is converted into oleic acid (-COOH), which no longer binds strongly to the particle surface and can be removed during washing/centrifugation. At the same time, chloride ions can occupy or passivate some surface sites, and the surface becomes more hydrophilic, allowing stable dispersion in water.

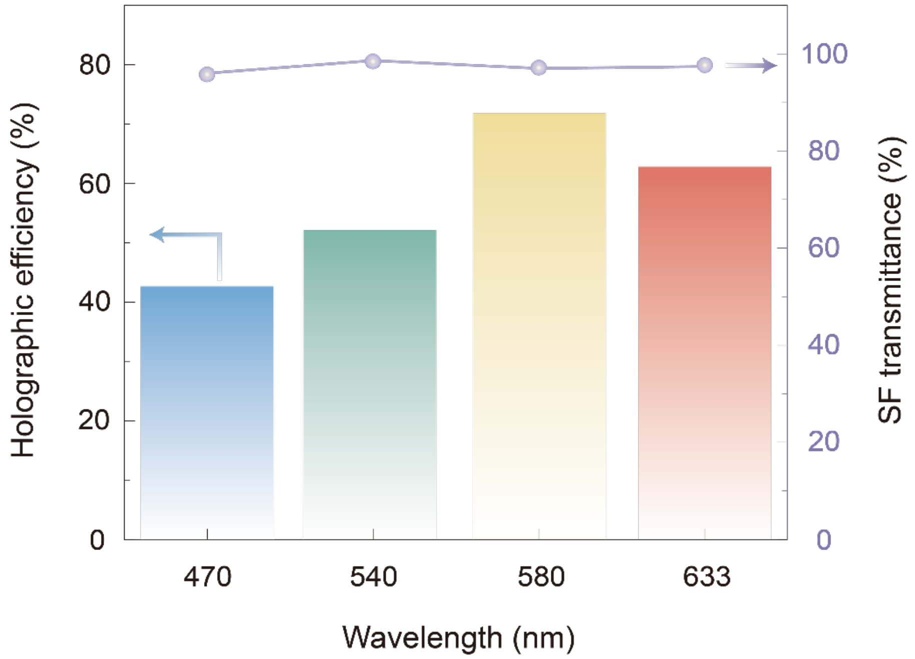

Fig. S13 Holographic efficiency of the LCN and transmittance of the SF film across different wavelengths. The LCN demonstrates a holographic efficiency that exceeds 40% over a broad spectral range, while the SF film, with its ultra-high transmittance (~97%), has a negligible effect on the holographic projection from the LCN.

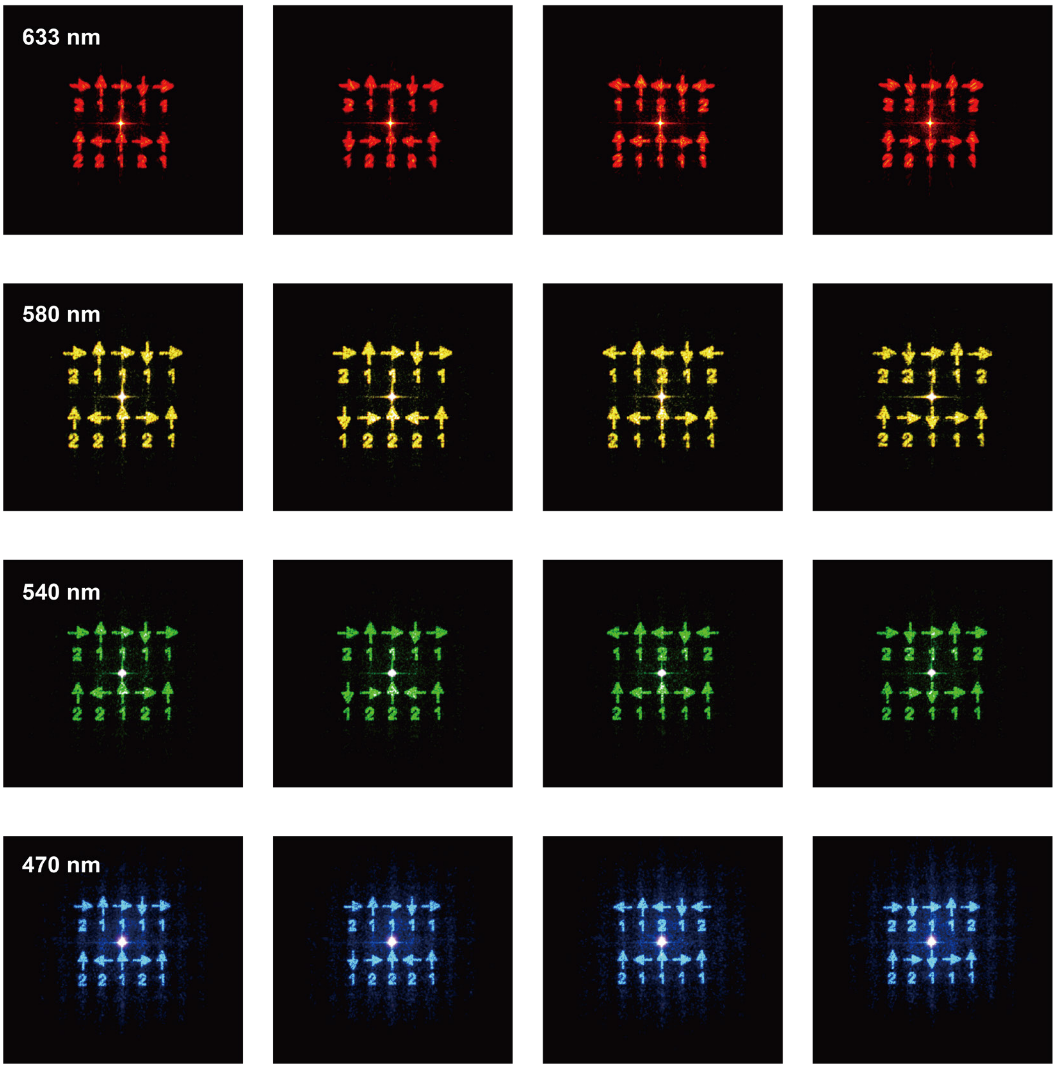

Fig. S14 Holographic commands extracted across a broad spectral range, demonstrating their high-fidelity display performance.

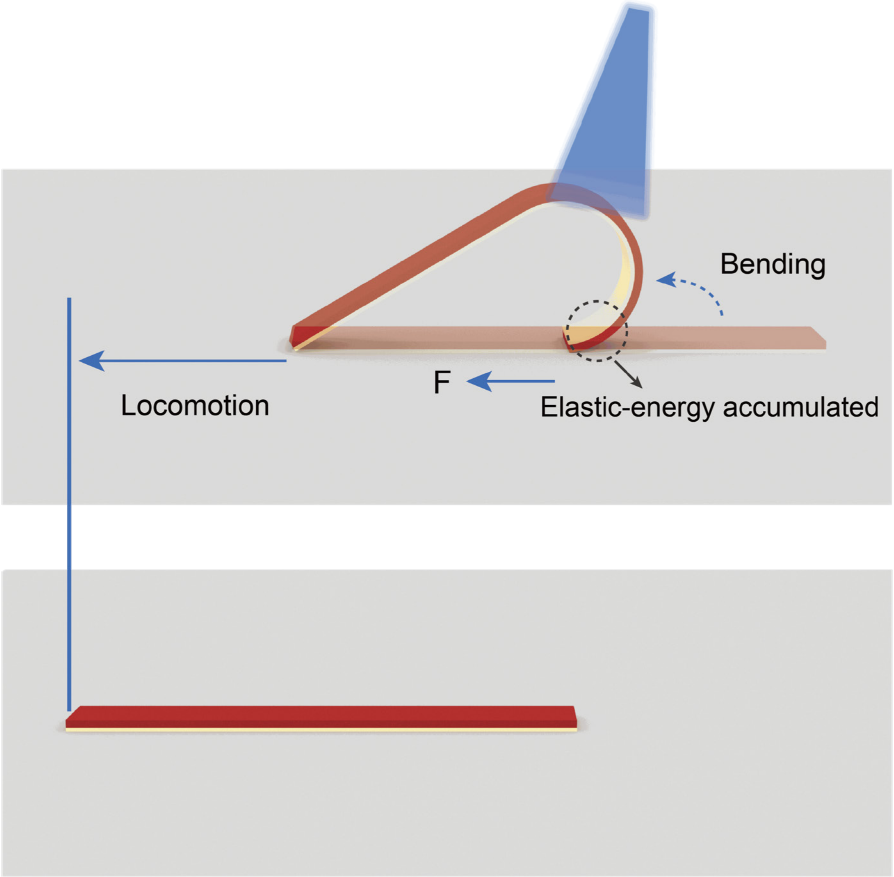

Fig. S15 Schematic illustration of the actuation mechanism underlying the locomotion of the LCN/SF bilayer actuator. Localized light irradiation near one end of the LCN/SF bilayer strip induces bending and arching at the irradiated region, which subsequently drives directional motion toward the opposite end through the release of stored elastic energy.

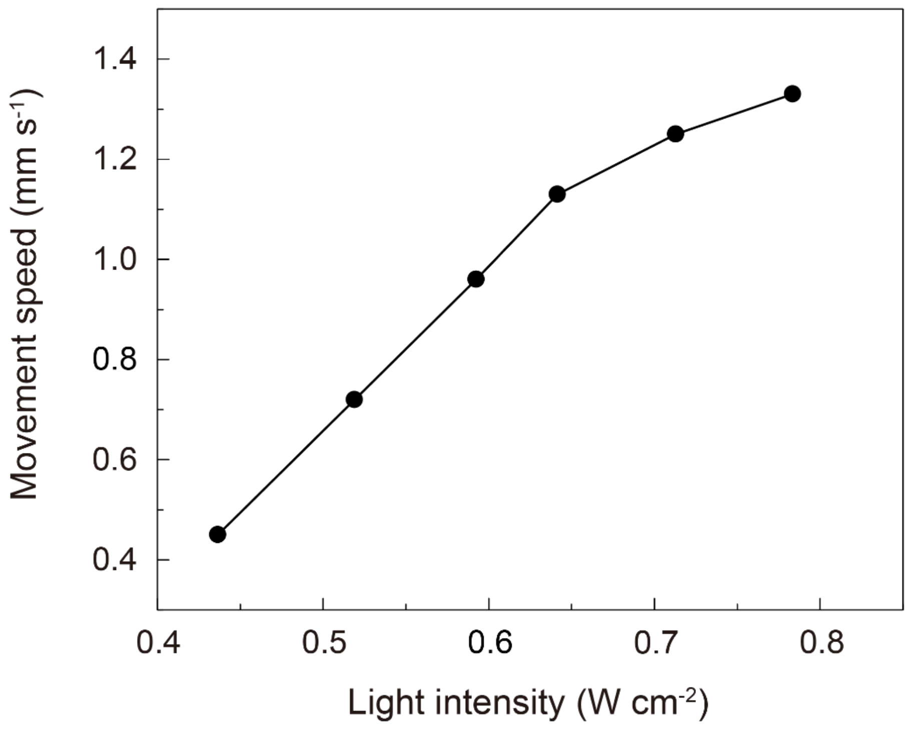

Fig. S16 The curve of the walking robot's movement speed as a function of light intensity.

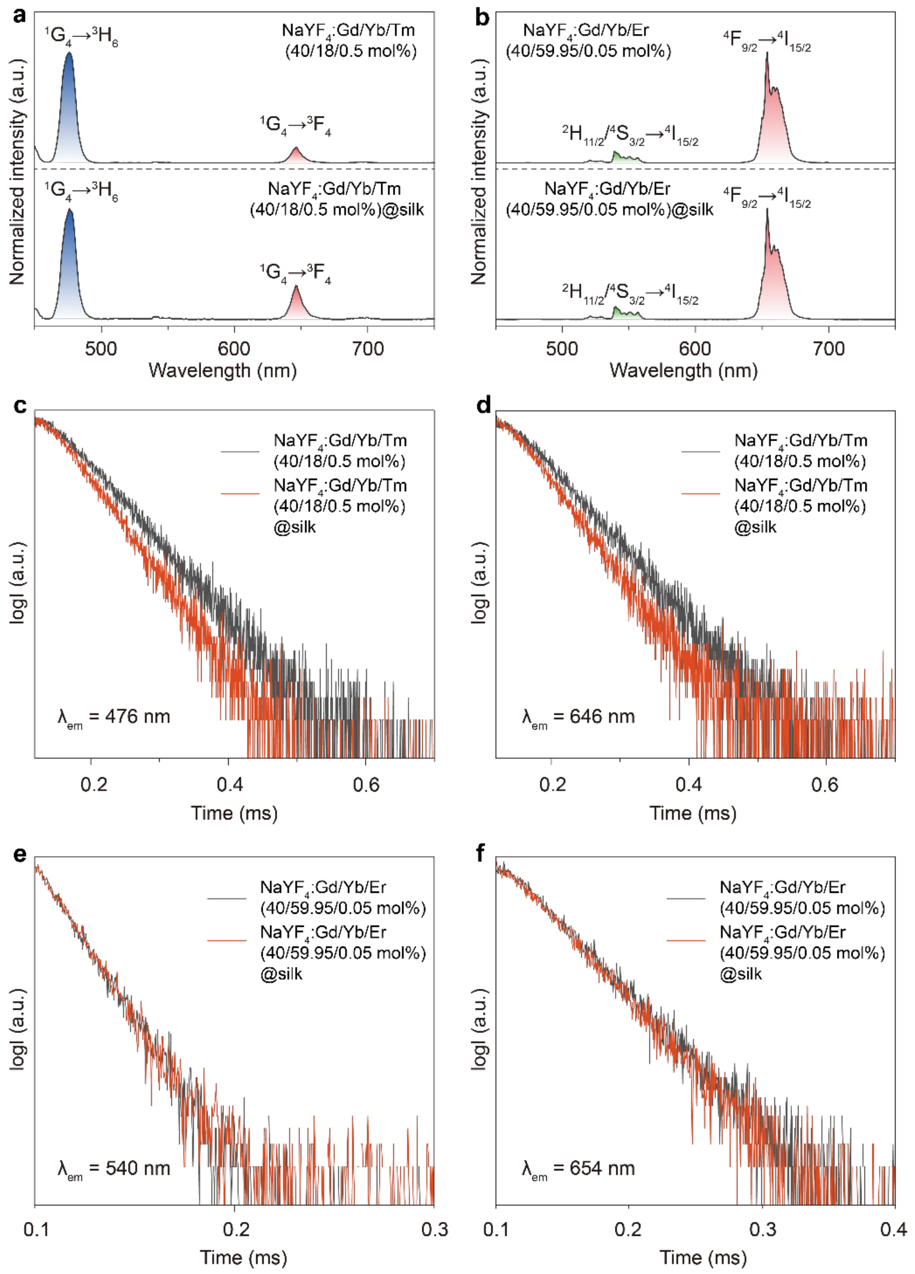

Fig. S17 Luminescent spectra and decay curves of the UCNPs before and after embedding within SF matrix. (a) UCL spectra of NaYF4:Gd/Yb/Tm (40/18/0.5 mol%) and NaYF4:Gd/Yb/Tm (40/18/0.5 mol%)@silk. (b) UCL spectra of NaYF4:Gd/Yb/Er (40/59.95/0.05 mol%) and NaYF4:Gd/Yb/Er (40/59.95/0.05 mol%)@silk. Decay curves of UCL of NaYF4:Gd/Yb/Tm (40/18/0.5 mol%) and NaYF4:Gd/Yb/Tm (40/18/0.5 mol%)@silk at (c) 476 nm and (d) 646 nm. Decay curves

of UCL of $NaYF_4$:Gd/Yb/Er (40/59.95/0.05 mol%) and $NaYF_4$:Gd/Yb/Er (40/59.95/0.05 mol%)@silk at (e) 540 nm and (f) 654 nm.

Table S1. Actuation performance comparison of LCN/SF with other reported humidity and photothermal actuators.

**Materials**	**Photothermal actuation**		**Humidity-driven actuation**		**Programmable shape morphing and multimodal actuation**	**Reference**
	**maximum bending angle/curvature**	**response time**	**maximum bending angle/curvature**	**response time**		
LCN/Silk	135°/1.58 cm^{-1} (0.59 W cm^{-2})	12 s	228.71°/2.03 cm^{-1} (RH = 85%)	180 s	Yes	This work
MP/CP	1128° (0.2 W cm^{-2})	10 s	516° (RH = 90%)/	0.5 s	No	[1]
CINPs/CNFs-PLA	2.28 cm^{-1} (0.6 W cm^{-2})	10 s	6.28 cm^{-1} (RH = 97%)	200 s	No	[2]
CNT–Nafion/PE	1.12 cm^{-1} (0.1 W cm^{-2})	10 s	5.24 cm^{-1} (RH = 80%)	/	No	[3]
PEO/MOF/PVDF/Mxene	7.21 cm^{-1} (0.1 W cm^{-2})	21 s	7.21 cm^{-1} (RH = 95%)	/	No	[4]

LCNs	360° (0.1 W cm^{-2})	/	180° (RH = 80%)	/	No	[5]
Mxene	112° (0.23 W cm^{-2})	8 s	150° (RH = 100%)	20 s	No	[6]
PPy@G-BC/BOPP	1.18 cm^{-1} (0.3 W cm^{-2})	/	1.26 cm^{-1} (RH = 90%)	/	No	[7]
GO/PPy	1.40 cm^{-1} (0.08 W cm^{-2})	3 s	4.18 cm^{-1} (RH = 70%)	9 s	No	[8]
CNP/rGO	67° (0.16 W cm^{-2})	7 s	90° (RH = 100%)	9 s	No	[9]
RGO/GO	0.51 cm^{-1} (0.3 W cm^{-2})	16 s	0.38 cm^{-1} (RH = 77%)	45 s	No	[10]

Note S1

Explanation of the image-forming principle of holography

Computer-generated holography inherits the advantage of traditional optical holography in reconstructing true 3D complex-amplitude wavefronts through diffraction, while transforming the cumbersome experimental operations of optical holography into three processes: wavefront computation, wavefront encoding, and wavefront reconstruction.

Wavefront computation refers to the numerical propagation of the object wavefront using matrix-based calculations, thereby obtaining the mathematical description of the complex-amplitude wavefront on the hologram plane.

Wavefront encoding converts the complex-amplitude distribution on the hologram plane into a computer-generated hologram that matches the numerical format of the display medium.

Wavefront reconstruction denotes the optical reconstruction process of the computer-generated hologram: the hologram is uploaded onto a wavefront modulation device, and under coherent light illumination, the diffraction propagation reconstructs the object wavefront.

By simulating the holographic recording process with computers, computer-generated holography greatly simplifies the generation of holograms. In this work, we adopt an improved Gerchberg–Saxton (GS) algorithm: taking as input the target far-field holographic image and the amplitude distribution of the near-field input beam (here chosen as a Gaussian beam), the algorithm performs iterative optimization with the

introduction of random perturbations to obtain a high-quality near-field phase map φ_3. By simply modulating the incident Gaussian beam with the calculated phase distribution φ_3, the designed arbitrary holographic image can be produced in the far field. The experimental setup for the extraction of holographic information is shown in Fig. S10. Meanwhile, the specific flow chart of the Gerchberg-Saxton algorithm for LCN holography is shown in Fig. S11.

Note S2

The mechanism of LCN with embedded holograms for holographic imaging

The optical transmission matrix expression of the LCN device is:

$$T(\alpha) = \mathrm{R}(\alpha)\begin{bmatrix} e^{-i\frac{\Gamma}{2}} & 0 \\ 0 & e^{i\frac{\Gamma}{2}} \end{bmatrix}\mathrm{R}(-\alpha)$$

Here, α denotes the in-plane orientation angle of the LC director, R(α) is the rotation matrix, and Γ represents the phase retardation between the ordinary and extraordinary waves. The incident field E_{in}, chosen here as circularly polarized (CP) light, is related to the output optical field of the LCN as follows:

$$E_{\mathrm{out}} = T(\alpha)E_{\mathrm{in}} = T(\theta)[1 \ \pm \mathrm{i}]^{\mathrm{T}} = \left(\frac{e^{-i\frac{\Gamma}{2}}+e^{i\frac{\Gamma}{2}}}{2}\right)[1 \ \pm \mathrm{i}]^{\mathrm{T}} + \left(\frac{e^{-i\frac{\Gamma}{2}}-e^{i\frac{\Gamma}{2}}}{2}\right)e^{i(\pm 2\theta)}[1 \ \mp \mathrm{i}]^{\mathrm{T}}$$

It converts the incident CP light into the opposite helicity with geometric phases $\mathrm{e}^{+\mathrm{i}2\alpha}$ ($|\mathbf{R}\rangle \rightarrow \mathrm{e}^{+\mathrm{i}2\alpha}|\mathbf{L}\rangle$ and $|\mathbf{L}\rangle \rightarrow \mathrm{e}^{-\mathrm{i}2\alpha}|\mathbf{R}\rangle$, where $|\mathbf{L}\rangle$ and $|\mathbf{R}\rangle$ represent the left and right spin states of light). The acquired geometric phases show a linear relation of twice of α, where $|\mathbf{L}\rangle = \frac{\sqrt{2}}{2}[1 \ -\mathrm{i}]^{\mathrm{T}}$ and $|\mathbf{R}\rangle = \frac{\sqrt{2}}{2}[1 \ +\mathrm{i}]^{\mathrm{T}}$. Therefore, by employing photoalignment technology, we realize an in-plane orientation angle distribution of the LCN as $\varphi_3/2$. With a CP Gaussian beam incident on the device, the designed far-field holographic image can then be generated.

Note S3

The mechanism of DMD-assisted photopatterning of LCN

To achieve pixelated LC superstructures with high quality, we delicately upgrade the photoalignment technique by carefully aligning the optical path of the home-made dynamic microlithography system and optimizing the exposure parameters during the photopatterning process, including the exposure location, exposure intensity, and exposure time. The metal-wire nanograting is used for an increased degree of linear polarization (>7000:1); the uniformity of the light source is improved (≥ 95%), and the focusing precision is measured as 1 μm. As a result, the spatial resolution of 1.1 μm is achieved, and the precision of the polarization angle is reduced to ± 0.2°.

The LC director distribution for LCN-holography is generated by our modified GS algorithm. Detailed photopatterning processes are provided as follows. The empty LC cell is placed at the image plane of the digital micromirror device-based microlithography system to record the patterns of LC director distribution via a multistep, partly overlapping exposure process with synchronous polarization control. The calculated director distribution varying from 0 to π is replaced by 36 subregions equally, and each subregion is endowed with a uniform director value from $\pi/36$ to π in an interval of $\pi/36$. This determines the exposure location. A sum of five adjacent subregions (the sum-region) is exposed simultaneously with the exposure intensity of 0.8 mW/cm^2, exposure wavelength of 405 nm, and exposure time of 3.0 s, which is insufficient to induce a stable reorientation of SD1. The subsequent exposure of the sum-region shifts one subregion with the polarizer rotating 5° synchronously. Finally, each subregion is exposed five times with a total exposure time of 15.0 s, which is

enough to reorient the SD1 molecules, which locally control the LC molecules to form a hologram with space-variant azimuthal orientations.

Note S4

The mechanism of UCNPs' emission colors tuning

The observed color change mainly comes from a change in the relative intensity of Er^{3+} green and red transitions, i.e., the red-to-green (R/G) ratio, which is strongly affected by the Yb-to-Er energy transfer efficiency and the relaxation pathways inside Er^{3+}. Under 980 nm excitation, Yb^{3+} acts as a sensitizer and absorbs the pump light efficiently, then transfers energy to Er^{3+}. After sequential energy transfer steps, Er^{3+} can populate higher excited states and emit green light from the $^2H_{11/2}$/$^4S_{3/2}$→$^4I_{15/2}$ transitions. Er^{3+} can also emit red light from the $^4F_{9/2}$→$^4I_{15/2}$ transition (around 650 nm). The key point is that the $^4F_{9/2}$ red level is not only reached directly from higher Er^{3+} states, but it can also be fed efficiently through non-radiative relaxation from the green levels and through energy-transfer routes involving the intermediate $^4I_{13/2}$ level. Therefore, when we change the Yb/Er ratio, we change how fast Er^{3+} is pumped and how the population is redistributed between the green and red emitting levels, which finally changes the R/G ratio and the apparent emission color.

In our samples, the composition with a moderate Yb content and a typical Er content, $NaYF_4$: Gd/Yb/Er (40/18/2 mol%), shows mainly green emission. In this case, the energy transfer from Yb^{3+} to Er^{3+} is sufficient to populate the green emitting levels, and the green channels remain strong, so the overall emission looks green. When we increase the Yb content while keeping Er at 2 mol% ($NaYF_4$: Gd/Yb/Er (40/58/2 mol%)), Yb^{3+} absorbs more pump photons and transfers more energy to Er^{3+}. This increases the population of intermediate and higher Er^{3+} levels and strengthens the pathways that feed the $^4F_{9/2}$ red level (for example, relaxation from green levels to $^4F_{9/2}$,

and feeding of $^4F_{9/2}$ through the $^4I_{13/2}$ reservoir). As a result, the red component becomes stronger relative to the green component, so the emission becomes a mixed color and appears yellow. When the Yb/Er ratio becomes extremely high ($NaYF_4$: Gd/Yb/Er (40/59.95/0.05 mol%)), the number of Er^{3+} activators is very small, while many Yb^{3+} sensitizers surround each Er^{3+}. In this situation, each Er^{3+} can receive energy very efficiently from nearby Yb^{3+}, but the population distribution can shift because the high-energy green emitting levels are generally more sensitive to non-radiative loss (for example, relaxation assisted by the local environment and surface/interface effects), while the lower-energy $^4F_{9/2}$ red level is relatively less sensitive. In addition, the $^4I_{13/2}$ intermediate level can act as a long-lived “storage” level and can be further pumped to feed $^4F_{9/2}$, which also favors red emission. Therefore, the red channel becomes dominant and the overall emission appears red.

Note S5

Analysis of the effect of SF matrix on UCNPs' luminescent properties.

Experimentally, we observe that embedding in SF does change the intensity ratios and lifetimes, but the effect depends on the dopant system. For $NaYF_4$:Gd/Yb/Tm (40/18/0.5 mol%), the red-to-blue emission ratio becomes larger after embedding in the SF film (Fig. S17a). At the same time, the lifetimes decrease at both monitored wavelengths: the 480 nm lifetime decreases from 759.8407 μs (UCNPs) to 605.4951 μs (UCNP-SF film), and the 646 nm lifetime decreases from 763.4440 μs to 607.1123 μs (Fig. S17c and d). Since the SF film does not absorb in the visible, the observed change in the red/blue ratio is most reasonably explained by matrix/interface effects (and possible wavelength-dependent scattering/light outcoupling in the solid film), rather than by visible reabsorption in SF. For $NaYF_4$:Gd/Yb/Er (40/59.95/0.05 mol%), the red-to-green emission ratio becomes smaller after embedding (Fig. S17b). The green lifetime at 540 nm remains almost unchanged (174.4264 μs→173.3008 μs), while the red lifetime at 654 nm shows a modest decrease (404.1938 μs→388.6645 μs) (Fig. S17e and f). This suggests that SF embedding has only a small impact on Er^{3+} upconversion dynamics overall, but the red channel is slightly more sensitive, consistent with the fact that the red emission has a longer intrinsic lifetime and can show a more visible change when a small additional non-radiative decay channel is introduced by the matrix/interface.

Note S6

Explanations regarding the functions of the four petals and the entire encryption process of the walking robot

All four petals are composed of LCN/SF bilayer structures, with the SF film layer doped with UCNPs. By tuning the doping ratios of Yb^{3+}, Er^{3+}, and Tm^{3+} ions, different fluorescence emission colors can be achieved. For the Yb^{3+}/Er^{3+} co-doped system, emission peaks appear at both 650 nm and 540 nm. By adjusting the relative doping ratios of these two ions, three visually distinct fluorescence colors—red, yellow, and green—can be obtained. For the Yb^{3+}/Tm^{3+} co-doped system, the emission intensity of Tm^{3+} at 480 nm is much stronger than that at 650 nm, resulting in blue upconversion luminescence. As stated in the main text under the section "*Optically Informative Module*", the specific ion-doping ratios for the four different emission colors are: blue (Yb/Tm: 18/0.5 mol%), green (Yb/Er: 18/2 mol%), yellow (Yb/Er: 50/1 mol%), red (Yb/Er: 80/0.05 mol%). It is worth noting that, as transparent media, UCNPs with different dopants embedded in the SF films do not alter their appearance or transparency, which is essential for optical encryption applications.

In the design, we deliberately defined a one-to-one positional correspondence between the four petals and the four phase holograms on the base. After identifying the different fluorescence colors of the four petals, we used a supercontinuum laser with wavelengths of 633 nm (red), 580 nm (yellow), 540 nm (green), and 480 nm (blue) to ensure that the holograms would also exhibit four corresponding colors. Thus, the hologram colors were visually correlated with the fluorescence colors of the petals. In fact, a single-wavelength supercontinuum laser can extract four distinct holographic information sets,

and we deliberately matched their colors for consistency. Different color combinations correspond to different sets of driving instructions, thereby enabling information encryption.

The entire encryption process is as follows: First, the four triangular bilayer films were shaped into closed petals and then assembled with the base containing the embedded phase holograms. Under high humidity, the petals unfold, and upon excitation with a 980 nm laser, each of the four petals exhibits a distinct fluorescence color. At this stage, the positions of the petals were carefully recorded to ensure that each petal corresponded to a phase hologram projection of the same color in the subsequent step. Next, the phase holograms at the bottom were illuminated using the supercontinuum laser, producing four different holographic images in red, yellow, green, and blue, which directly matched the fluorescence colors of the petals. Since there are four holograms in total, there exist $4^4 = 256$ possible reading sequences, with each sequence corresponding to a different set of motion instructions. However, only one unique correct order—RGYB—yields the complete maze-escape pathway, which then guides the subsequent actuation process.

Legends for Movies:

Movie S1. Light-responsive actuation of the LCN/SF bilayer actuator.

Movie S2. Humidity-responsive actuation of the LCN/SF bilayer actuator.

Movie S3. FEA simulations of the programmable deformations under 488 nm light.

Movie S4. Light-actuated bending of the gripper arms.

Movie S5. Soft robotic gripper carrying holographic instructions for object sorting.

Movie S6. Maze navigation based on the specific holographic path-guiding instructions.

References:

[1] Wang, Y. C. et al. Ultrafast and Multi-Stimuli-Responsive MXene Soft Actuators via Heterostructure Design for Biomimetic Applications. *Adv. Funct. Mater.* **36**, e14386 (2026).

[2] Chen, Z. et al. Multistimuli-Responsive Actuators Derived from Natural Materials for Entirely Biodegradable and Programmable Untethered Soft Robots. *ACS Nano* **17**, 23032−23045 (2023).

[3] Chang, L. F. et al. A Versatile Ionomer-Based Soft Actuator with Multi-Stimulus Responses, Self-Sustainable Locomotion, and Photoelectric Conversion. *Adv. Funct. Mater.* **33**, 2212341 (2023).

[4] Zhang, Y. F. et al. Highly-Aligned All-Fiber Actuator with Asymmetric Photothermal-Humidity Response and Autonomous Perceptivity. *Adv. Mater.* **36**, 2404696 (2024).

[5] Wani, O. M., Verpaalen, R., Zeng, H., Priimagi, A. & Schenning, A. P. H. J. An Artificial Nocturnal Flower via Humidity-Gated Photoactuation in Liquid Crystal Networks. *Adv. Mater.* **31**, 1805985 (2019).

[6] Ma, J. N. et al. Multiresponsive MXene Actuators with Asymmetric Quantum-Confined Superfluidic Structures. *Adv. Funct. Mater.* **34**, 2308317 (2024).

[7] Weng, M. C. et al. Multi-Functional Actuators Made with Biomass-Based Graphene-Polymer Films for Intelligent Gesture Recognition and Multi-Mode Self-Powered Sensing. *Adv. Sci.* **11**, 2309846 (2024).

[8] Dong, Y. et al. Multi-stimuli-responsive programmable biomimetic

actuator. *Nat. Commun.* **10**, 4087 (2019).

[9] Che, X. P. et al. Bio-inspired water resistant and fast multi-responsive Janus actuator assembled by cellulose nanopaper and graphene with lignin adhesion. *Chem. Eng. J.* **433**, 133672 (2022).

[10] Ma, J. N. et al. Laser Programmable Patterning of RGO/GO Janus Paper for Multiresponsive Actuators. *Adv. Mater. Technol.* **4**, 1900554 (2019).
